# In Vivo and In Vitro Characterization of the RNA Binding Capacity of SETD1A (KMT2F)

**DOI:** 10.3390/ijms242216032

**Published:** 2023-11-07

**Authors:** Harem Muhamad Amin, Beata Szabo, Rawan Abukhairan, Andras Zeke, József Kardos, Eva Schad, Agnes Tantos

**Affiliations:** 1 Institute of Enzymology, HUN-REN Research Centre for Natural Sciences, H-1117 Budapest, Hungary; harem.muhamad@univsul.edu.iq (H.M.A.); szabo.beata@ttk.hu (B.S.); rawan.kabu@gmail.com (R.A.); schad.eva@ttk.hu (E.S.); 2 Doctoral School of Biology, Institute of Biology, ELTE Eötvös Loránd University, H-1117 Budapest, Hungary; 3 Department of Biology, College of Science, University of Sulaimani, Sulaymaniyah 46001, Kurdistan Region, Iraq; 4 ELTE NAP Neuroimmunology Research Group, Department of Biochemistry, Institute of Biology, ELTE Eötvös Loránd University, H-1117 Budapest, Hungary; kardos@elte.hu

**Keywords:** SETD1A, KMT2F, RRM domain, histone lysine methyltransferase, RNA binding, non-coding RNA, microscale thermophoresis, RNA immunoprecipitation

## Abstract

For several histone lysine methyltransferases (HKMTs), RNA binding has been already shown to be a functionally relevant feature, but detailed information on the RNA interactome of these proteins is not always known. Of the six human KMT2 proteins responsible for the methylation of the H3K4 residue, two—SETD1A and SETD1B—contain RNA recognition domains (RRMs). Here we investigated the RNA binding capacity of SETD1A and identified a broad range of interacting RNAs within HEK293T cells. Our analysis revealed that similar to yeast Set1, SETD1A is also capable of binding several coding and non-coding RNAs, including RNA species related to RNA processing. We also show direct RNA binding activity of the individual RRM domain in vitro, which is in contrast with the RRM domain found in yeast Set1. Structural modeling revealed important details on the possible RNA recognition mode of SETD1A and highlighted some fundamental differences between SETD1A and Set1, explaining the differences in the RNA binding capacity of their respective RRMs.

## 1. Introduction

Histone modifications are finely tuned molecular mechanisms by which many basic nuclear processes are controlled. These epigenetic marks are placed on specific residues in the disordered histone tails by designated enzymes and collectively create an intricate regulatory pattern responsible for the implementation of complete genetic programs. One of the most prevalent histone modifications is lysine methylation, which is carried out by histone lysine methyltransferases (HKMTs), a large group of enzymes with more than fifty members in the human genome, each responsible for the placement of a specific histone lysine mark. This modification in turn induces the recruitment of transcription factors or directly influences chromatin structure, thus achieving alterations in gene expression. Proteins with histone lysine methylation activity all contain a SET domain—with the exception of DOT1L—which is responsible for the catalytic activity [1].

H3K4 methylation is carried out by a single Set1 protein in yeast, and by three family members in Drosophila: Trx (Trithorax), Trr (Trithorax related), and dSet [1]. In mammals, two orthologs are found for each Drosophila H3K4 methyltransferase: KMT2A and B (MLL1/2) are related to Trx, KMT2C and D (MLL3/4) to Trr, and KMT2E and F (SETD1A/B) to dSet [2]. The proteins themselves exhibit moderate enzymatic activity and require the interaction with other proteins to achieve their full methyltransferase capacity [3]. WDR5, RbBP5, Ash2L, and Dpy30 proteins form the core components of this active COMPASS (Complex of Proteins Associated with Set1) complex, but other, complex-specific subunits with regulatory functions are also known [4]. The six human KMT2 proteins have similar histone methyltransferase activities in pairs, with KMT2A and B carrying out H3K4 trimethylation required for *HOX* gene transcription [5], KMT2C and D responsible for H3K4 monomethylation in enhancer regions, and KMT2F (SETD1A) and G (SETD1B) performing the majority of global H3K4 di- and trimethylation [6]. Importantly, their functions are non-redundant, as they cannot compensate for the loss of their methyltransferase pair and have different roles in the regulation of development, which is also mirrored by their unique domain structure [3] beyond the universal SET domain. These specific differences between these proteins suggest the existence of unique roles which are not related to histone methylation and several such functions have already been experimentally confirmed [3] in the case of KMT2A [7], KMT2C [8], KMT2D [9], SETD1A and SETD1B [10].

The different functions are especially evident for SETD1A and SETD1B, which show markedly different intracellular localization [11], already suggesting alterations in the interactome and regulation. Both are indispensable for early embryonic development in mice, but SETD1A is required before gastrulation, while SETD1B is necessary at a later state [10]. Of the two proteins, only SETD1A is essential for embryonic stem cell viability [10,12] and overexpression of SETD1B is insufficient to rescue the *SETD1A* knockout cells [10]. Even in adulthood, both proteins appear to be essential, but have specific functions, as the deletion of *SETD1A* leads to death in 3 weeks, but *SETD1B* knockout mice survive for 30 weeks [13].

In humans, SETD1A is increasingly reported to be related to the development of schizophrenia, as several studies identified mutations in the *Setd1A* gene in schizophrenia patients [13,14]. Interestingly, these mutations were located in regions outside of the SET domain, underlining their physiological importance. SETD1B, on the other hand, appears to be more involved in the development of autism and epilepsy [15], with mutations found both in and outside the SET domain [16,17]. Another example of the differences between the functions of the two paralogues is that SETD1A, but not SETD1B, is involved in DNA damage repair [18].

The molecular mechanisms that would explain these functional differences are poorly understood, but they are thought to originate from the specific functions of protein regions outside of the SET domain [3]. Therefore, it is imperative to study and describe the role of these segments for the understanding of the functions of KMT2 proteins in their full complexity.

SETD1A and SETD1B are unmatched in the family as they both contain canonical RNA binding (RRM) domains, originating from their evolutionary ancestor, also giving rise to the yeast Set1 protein (with two RRM domains arranged in tandem) [19] (Supplementary Figure S1). The structure and function of the RRM domain in Set1 and the importance of the RNA binding have been extensively studied and it was concluded that efficient RNA binding was achieved by the combination of the two separate RNA interacting domains [19], and RRM1 was incapable of recognizing RNA on its own. Later experiments confirmed that RNA binding performed a regulatory role in the function of Set1 [20] by directing the appropriate positioning of the enzyme along transcription units, but it was also clear that Set1 remained bound to RNAs even after transcription.

In the light of the importance of RNA binding in the regulation of Set1, it is surprising that the RRM domains and the RNA binding capacity of SETD1A and SETD1B have not yet been studied. Therefore, we set out to determine if the RRM domain of SETD1A is a functional RNA binding unit and whether RNA binding is a prominent feature of this protein.

## 2. Results

### 2.1. SETD1A Interacts with a Broad Range of Coding and Non-Coding RNAs

**Figure 1:**
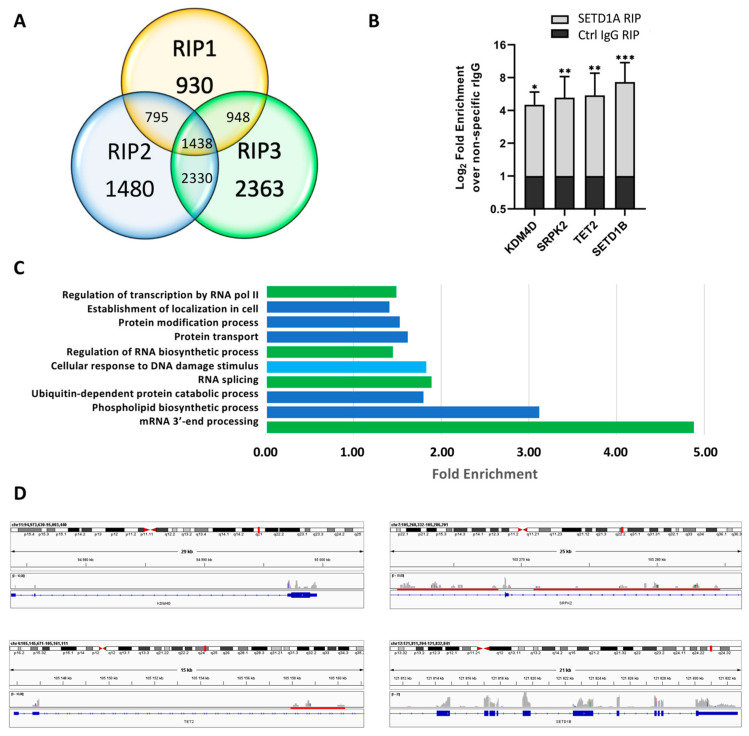
Analysis of the mRNAs associated with SETD1A. (**A**). Number of mRNAs that showed higher FPKM values than the negative control in the three independent RIP experiments. (**B**). RT-qPCR results of the selected mRNAs. Fold enrichment of immunoprecipitated RNAs was calculated as 2^(−ΔΔCt [SETD1A RIP/NS Rb IgG RIP])^. Mean values and standard errors of three independent biological replicas are shown. Statistical analysis was performed using one-way ANOVA; *, **, and *** indicate significant differences from non-specific rabbit IgG control samples at *p* < 0.05, *p* < 0.005, and *p* < 0.001 values, respectively. (**C**). PANTHER protein classes that were significantly enriched in the mRNAs present in all three RIP-seq datasets (FDR adjusted *p*-value < 0.05). Functional classes related to RNA transcription regulation and RNA processing are labeled green and the DNA damage response class is labeled light blue. (**D**). IGV views of the mapped NGS reads for the selected genes: *KDM4D* (Lysine-specific demethylase 4D), *SRPK2* (SRSF protein kinase 2), *TET2* (Methylcytosine dioxygenase TET2), and *SETD1B* (Histone-lysine N-methyltransferase SETD1B). Reads in the NGS data are represented by gray columns, and intronic regions are highlighted by red bars. Exons are represented by blue boxes.

**Figure 2:**
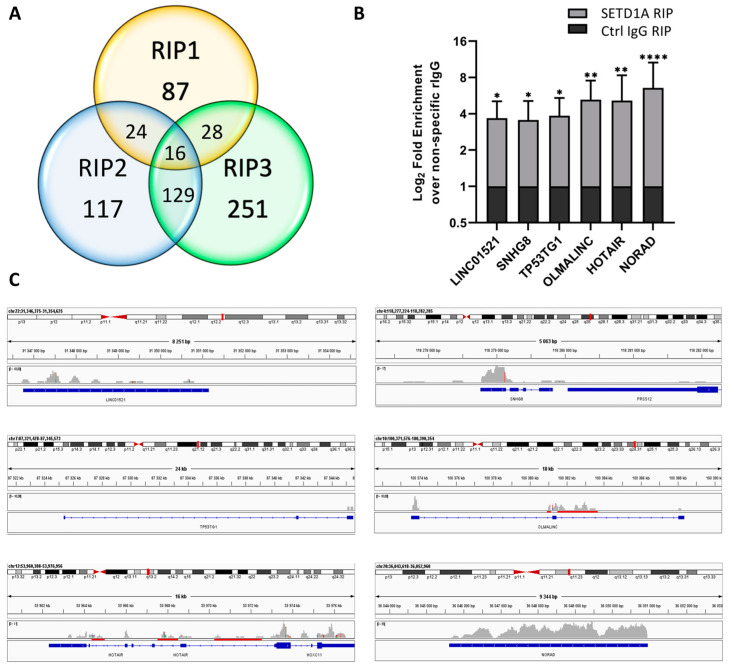
Analysis of the lncRNAs associated with SETD1A. (**A**). Number of lncRNAs that showed higher FPKM values than the negative control in the three independent RIP experiments. (**B**). RT-qPCR results of the selected lncRNAs. Fold enrichment of immunoprecipitated RNAs was calculated as 2^(−ΔΔCt [SETD1A RIP/NS Rb IgG RIP])^. Mean values and standard errors of three independent biological replicas are shown. Statistical analysis was performed using one-way ANOVA; *, **, and **** indicate significant differences from non-specific rabbit IgG control samples at *p* < 0.05, *p* < 0.005, and *p* < 0.0001 values, respectively. (**C**). IGV views of the mapped NGS reads for the selected genes: LINC01521, SNHG8 (Small Nuclear Host Gene 8), TP53TG1 (TP53 Target 1), OLMALINC (Oligodendrocyte Maturation-associated Long Intergenic Non-coding RNA), HOTAIR (HOX Transcript Antisense RNA), and NORAD (Non-coding RNA Activated by DNA Damage). Reads in the NGS data are represented by gray columns, and intronic regions are highlighted by red bars. Exons are represented by blue boxes.

As the RRM domains are specific to the Set1 type HKMTs, we aimed at identifying the range of RNAs possibly interacting with SETD1A. For this, we carried out RNA immunoprecipitation experiments and identified the immunoprecipitated transcripts by next generation sequencing (RIP-Seq). In the case of SETD1A, three independent experiments returned 10,284 mRNAs and 652 non-coding RNAs (ncRNAs) that showed higher FPKMs in the positive samples than in the negative controls in at least one dataset. A total of 1438 mRNAs and 16 lncRNAs were present in all three replicates (Figure 1A and Figure 2A and Supplementary Tables S1–S3), indicating a broad range of RNAs bound by SETD1A. Out of these hits, 463 mRNAs and 9 lncRNAs showed a more than two-fold enrichment above the negative control in at least two replicates.

As a confirmation of the SETD1A RIP-Seq experiments, we performed RIP-qPCR tests with four selected mRNAs. As shown in Figure 1B, all four RNAs were significantly enriched in the RIP samples of SETD1A compared to a non-specific control antibody, indicating a strong association of the protein with these mRNAs. We could determine a four-fold increase in *KDM4D* mRNA compared with the negative control, which is in line with the RIP-seq results, where the increase in FPKM values was above four in all three replicates. mRNAs of several other KDM proteins were also found in the RIP datasets (Supplementary Table S1), indicating a possible regulatory role for SETD1A in histone demethylation.

Although *SRPK2* mRNA was found to be enriched over the negative control in only one of the RIP-Seq samples with a two-fold excess, it showed a significant enrichment in the RIP-qPCR experiments (Figure 1B), indicating that even the weaker hits in the NGS data may represent specific binding events.

*TET2* mRNA was pulled down by SETD1A similarly to *KDM4D* mRNA, as it was also highly enriched in all RIP-Seq replicates, and in two of those the negative control did not contain this mRNA. Importantly, the mRNAs of the other two TET proteins were also identified as bound by SETD1A, although they were enriched only in two (TET1) or one (TET3) replicate (Supplementary Table S1).

The mRNA of *SETD1B* showed the highest enrichment in the RIP-qPCR experiments with fold-change values close to eight and it was also enriched in two replicates in the NGS datasets (Figure 1B and Supplementary Table S1). *SETD1A* mRNA was also enriched in two RIP-Seq replicates, along with several other SET and KMT proteins (Supplementary Table S1). Even if their enrichment was not extremely high, with fold-change values between two and four in most cases, their presence indicates a regulatory role for SETD1A in the expression of other histone modifiers. The enrichment of mRNAs of proteins related to post-translational modifications, such as histone methylation, can also be seen in the functional classification of the NGS hits (Figure 1C).

If we consider the larger dataset, with RNAs showing enrichment above the negative control in all three replicates, we find that functions related to RNA processing and the DNA damage response are overrepresented (Figure 1C and Supplementary Table S4), while genes involved in immune response are significantly depleted. Similar molecular functions are also enriched in the smaller dataset, although, due to the lower number of genes, these are not statistically significant. Based on the available scientific literature, involvement in RNA processing and DNA damage response are among the non-histone related functions suggested for SETD1A [21,22].

Previous observations regarding the RNA binding activity of Set1 [20] suggest that the protein might be involved in RNA metabolism which was indicated by the presence of splicing-related RNAs in its interactome and the dominant presence of intronic regions in the bound RNAs. According to our analysis, SETD1A interacts with spliceosomal RNAs, such as RNU1 and RNU4 (Supplementary Table S2), as well as with scaRNAs and snoRNAs, underlining the possible existence of a similar function. Nevertheless, when searching for intronic regions within the RNAs in the RIP datasets, we found several mRNAs that contained no reads in these segments (Figure 1D), indicating that SETD1A RNA binding can occur both co- and post-transcriptionally. Importantly, the mRNAs of SETD1B and other histone modifier proteins fall into the category with no bound intronic regions (Supplementary Figure S2), suggesting that these mRNAs interact with SETD1A in their mature form.

Similarly to several other HKMTs [19,23], SETD1A also binds several non-coding RNAs (Figure 2A and Supplementary Table S2).

Among the 16 lncRNAs enriched in all three independent RIP experiments, we can find highly conserved transcripts such as CHASERR that might play a role in transcription activation through interacting with nascent RNAs [24]: NRAV, which is involved in the regulation of immune responses and different cancers [25,26]; HCG11, which can act as a tumor suppressor [27] or an oncogene [28], depending on the interacting partners; and NORAD, which is also known to be involved in several different cancers [29,30].

The possible role of SETD1A in RNA processing was indicated by the presence of splicing related non-coding RNAs in the RIP datasets. RNU4-2, scaRNA5, and scaRNA7 were present in all three RIP replicates, while altogether 53 different RNU RNAs and 10 scaRNAs were identified in at least one RIP dataset (Supplementary Table S2) as being enriched over the negative control.

RIP-qPCR experiments confirmed the strong interaction of SETD1A with six different lncRNAs (Figure 2B), with NORAD showing the highest fold-change compared to the negative control despite the fact that in the NGS datasets, its fold enrichment exceeded four in only one replicate. The three other lncRNAs with high enrichment in the RIP-qPCR samples, HOTAIR, OLMALINC, and TP53TG1, were enriched in only two RIP-Seq replicates, but the fold-changes in their FPKM values were high, with OLMALINC and TP53TG1 having no reads in the negative control samples. Even though SNHG8 belongs to the least enriched group of RNAs in the RIP-qPCR experiments, its presence in all three RIP-Seq replicates and its high fold enrichment in two of those indicate that it is a stable interactor of SETD1A.

Similar to SRPK2, the LINC01521 RNA was also only present in one RIP-Seq replicate, but it was still significantly enriched in the RIP-qPCR experiments.

Several lncRNAs are similarly processed as mRNAs, as they contain various exons and can have multiple alternatively spliced variants. In order to determine whether identified lncRNAs interact with SETD1A after post-transcriptional processing, we mapped the reads in the NGS datasets on the gene bodies of the lncRNAs tested in the RIP-qPCR experiments (Figure 2C). LINC01521 and NORAD contain only one exon, and of the four other RNAs, two (OLMALINC and HOTAIR) contained reads in intronic regions, indicating that, similarly to mRNAs, SETD1A can bind lncRNAs both co- and post-transcriptionally.

### 2.2. The SETD1A RRM Domain Is an Independent RNA Recognition Unit

Since the homologous yeast Set1 has already been shown to bind RNAs, RNA interaction of SETD1A was anticipated. However, it is important to note that the RRM1 domain (structurally most similar to the RRM domain of human SETD1A) of the Set1 protein could not recognize RNAs in itself in vitro, but required the presence of a second RNA recognition domain for RNA interaction [19].

**Figure 3:**
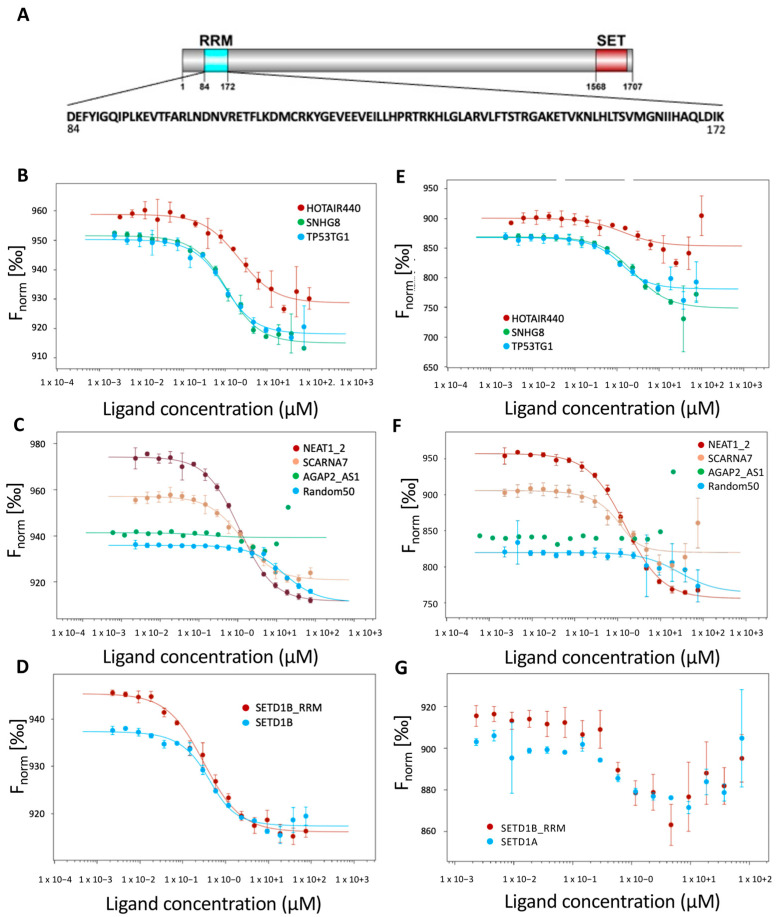
Binding of the SETD1A RRM domain to different RNAs. (**A**). Schematic representation of the domain structure of SETD1A (RRM domain—blue, SET domain—red), with the sequence of the RRM domain shown. (**B**,**C**). Binding curves of different lncRNAs and the SETD1A RRM domain measured with microscale thermophoresis T-jump (MST). RNAs that were confirmed to interact with SETD1A by RIP-qPCR are shown on panel (**B**). (HOTAIR440: red, SNHG8: green, TP53TG1: blue). RNAs detected only by RIP-seq (NEAT1_2: red, SCARNA7: beige, AGAP2_AS1: green) and the non-specific R50 (blue) are shown on panel (**C**). (**D**). Binding of the mRNAs of SETD1A (blue) and SETD1B (red) to SETD1A RRM domain measured by MST T-jump. (**E**,**F**). Binding curves of different ncRNAs and the SETD1A RRM domain measured with MST thermophoresis. (**E**). HOTAIR440 (red), SNHG8 (green), TP53TG1 (blue). (**F**). NEAT1_2 (red), SCARNA7 (beige), AGAP2_AS1 (green), R50 (blue). (**G**). Binding of the mRNAs of SETD1A (blue) and SETD1B (red) to SETD1A RRM domain measured by MST thermophoresis.

To determine whether the RRM domain of SETD1A is capable of RNA binding, we expressed the region between amino acids 89-197 of SETD1A (Figure 3A) and measured its interaction with different RNAs in vitro. Since our RIP-Seq data do not involve information on the exact binding site on the RNAs, we used longer RNA constructs for testing, opting for the full-length RNAs where possible. As the large number of identified RNAs suggested a rather non-specific binding, we selected a range of RNAs for the in vitro binding studies, covering RNAs that were highly enriched in the RIP experiments and others, which showed less prominent binding, as well as an artificial, random sequence RNA. The purified RRM domain proved to be stable in solution, and circular dichroism (CD) spectroscopy studies confirmed its folded structure (Supplementary Figure S3). Analysis for the secondary structure composition using the BeStSel method [31,32] revealed that the structure is rich in α-helix and also contains β-sheets, similarly to the crystal structure of the protein (PDB: 8ILY).

As a first approach, we used electrophoretic mobility shift essay (EMSA) experiments. These confirmed that the separated RRM domain is able to bind to all of the tested RNAs (Supplementary Figure S4), proving that it is a functional RNA binding unit even in the absence of other regions of SETD1A. In these experiments, the mobility shift that occurs upon protein–RNA binding indicates the interaction. In our experiments, all of the tested RNAs showed pronounced shifts towards higher weights (i.e., the combined weight of the RRM domain and the RNAs in a complex). In all cases, the highest, 10 μM RRM concentration resulted in the strongest mobility shift, while at lower RRM concentrations the RNAs showed specific differences. At 0.4 μM, only SNHG8 demonstrated significant interaction. Nevertheless, since the EMSA results are dependent on the size of the RNAs, these results cannot directly be used to compare the binding strength of the different RNA species.

While EMSA indicates binding events in general, it cannot describe fine differences in affinities of the different interactions. For further characterization of the RNA binding, we performed microscale thermophoresis (MST) measurements with a broader range of RNA partners. As shown on Figure 3B–G, the RRM domain of SETD1A could strongly bind most of the RNAs that were pulled down in the RIP experiments. The only exception was AGAP2-AS1, which showed no detectable interaction with the SETD1A RRM. Given that this lncRNA was identified as a SETD1A interactor in the NGS datasets, but with low fold-change values (Supplementary Table S2), this observation can indicate that it was a false-positive hit, or the possible existence of other RNA recognition elements in SETD1A.

MST can not only provide information on the binding affinities, it also bears information on different characteristics of the binding event.

Determining the behavior of the MST signal at different points during the measurement offers an opportunity to describe several events related to the interactions [33]. The first event in the MST measurement is the temperature jump (T-jump), which occurs immediately after turning on the laser. Since the sudden change in the fluorescence of the dye is dependent on its local molecular environment, the T-jump provides information on binding events that take place in the close vicinity of the dye, typically between 1 and 2 nm. Consistent changes in the T-jump of all binding RNAs (Figure 3B–D) clearly indicate a change in the local environment of the dye, confirming that the RNAs and the RRM are closely associated in the experiment, but because the RNAs are uniformly labeled through their sequences, the exact binding site cannot be determined.

The second event, thermophoresis, is related to the thermophoretic mobility and the diffusion capacity of the molecules studied, and therefore is sensitive to the size, charge, and the solvation of the molecule, providing information on the global changes that occur upon binding. This also indicates stable association of the RNAs and the RRM domain (Figure 3E–G), but in the case of the SETD1A and SETD1B mRNAs, the signals became unstable in higher protein concentration ranges (Figure 3G). This behavior is generally observed when larger particles appear in the system and is considered a sign of protein aggregation. Since similar behavior was not routinely observed in the same protein concentration range during the binding studies with other RNAs, this might stem from large-scale reorganization of the RNAs rather than the inherent behavior of the RRM domain.

The third event, back diffusion, occurs after the laser is switched off and is directly dependent on the size of the molecules in the system. If the binding resulted in a size change of the labeled partner, a concentration-dependent difference will be detectable in this phase of the MST, as it was also observed in our case (Supplementary Figure S5A–C).

The binding affinities determined by the three approaches are in good correlation for most of the RNAs, except for SCARNA7 and the two mRNAs (Table 1). Based on these, SETD1A RRM binds to the selected RNAs in the low micromolar range and appears to show high affinity to its own mRNA. The binding affinity does not seem to be directly affected by the length of the RNA, as RNAs with highly different lengths (SETD1A and SETD1B-RRM) are bound with similar strengths. It is also apparent that the interaction is not driven by the polyA tail, as the synthesized AGAP2-AS1 RNA contains a polyA sequence, yet it does not show measurable binding (for the RNA sequences, see Appendix A).

SCARNA7 yielded a higher affinity when the K_d_ was determined by the MST thermophoresis than the other two readings, indicating that, in this case, the overall behavior of the molecule is more affected by the binding. Since the K_d_ calculated on the basis of back diffusion is closer to the K_d_ based on the T-jump, this change is not directly related to the increased size of the complex, but is rather caused by the alterations in the hydration and/or charge brought about by the interaction.

The binding of the SETD1A RRM to the mRNAs of its own gene and that of its close homologue SETD1B resulted in an irregular behavior during the thermophoresis phase of the measurement (Figure 3G and Supplementary Figure S5D), preventing the calculation of a binding constant (Table 1) based on this parameter.

As mentioned above, the back diffusion phase of the MST curve indicates if there is an increase in size after the interaction of the investigated partners. In our case, we expect a significant increase in size, since the size of the RRM-RNA complexes should exceed that of the RNAs alone. The K_d_ values calculated based on the back diffusion phase of the MST show that this happened with all RNAs tested, except AGAP2_AS1 (Table 1), which also did not show any binding here.

### 2.3. Possible RNA-Interacting Interface of SED1A RRM

Based on the previously introduced results, we can assume that contrary to yeast Set1, the single RRM domain of SETD1A is a functional RNA-binding unit on its own, even if the existence of other, additional RNA recognition surfaces on the protein cannot be ruled out. This difference can only be understood by the comparison of the structures of the RRM domains. A recently published crystal structure of the RRM domain of SETD1A (PDB ID 8ILY) [34] allowed us to assess the hypothetical molecular mechanisms of RNA recognition of SETD1A.

**Figure 4:**
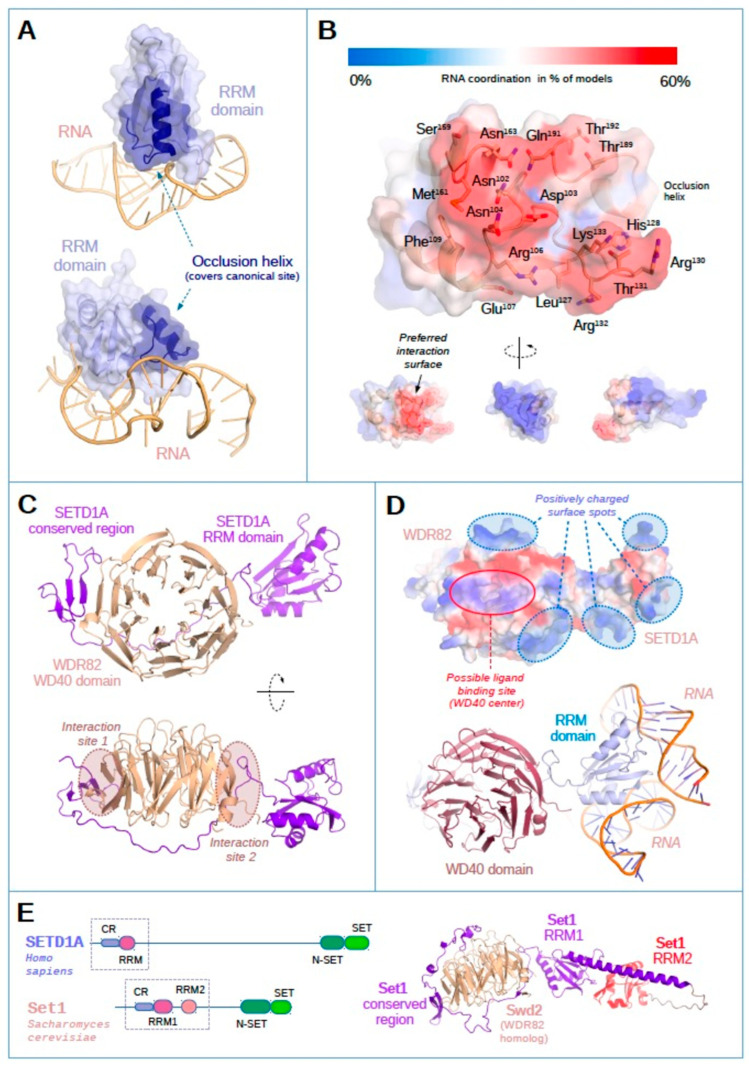
(**A**). Structural analysis of the RRM domain of human SETD1A (blue, with transparent surface), docked with two random RNA molecules. The occlusion helix (dark blue) covers the surface involved in RNA coordination in canonical RRM domains, yet it possibly also contributes to the generation of a novel RNA binding surface. (**B**). The majority of the simulated RNA binding events occur on a relatively broad surface of the RRM domain (with certain residues involved in RNA coordination in up to 60% of simulations). (**C**). The complex between the N-terminal region of SETD1A (residues 15–200, purple) and WDR82 (full length, wheat color), as modeled by AlphaFold2 multimer. (**D**). Surface charge character of the complex of SETD1A RRM/WDR82. Positive charge character is represented by blue and negative charge character is represented by red coloring. Bottom panel shows the superposition of two RNA-bound RRM models with the complex. (**E**). Schematic representation of the domain structures of human SETD1A and yeast Set1, with the main, RNA-interacting modules enframed. CR (Conserved Region) indicates the conserved interaction sites for WDR82 (SETD1A) and Swd2 (Set1) partner proteins. AlphaFold model of the complex between yeast Set1 and Swd2 is shown on the right.

According to the published structures, the RRM domains of SETD1A, SETD1B, and the homologous first RRM1 domain in yeast Set1 all contain an extra α-helix at the C-terminal region of the domain, which is not present in other canonical RRM domains [19,34]. This helix largely occludes the traditional RNA binding surfaces on the central β-sheets of the RRMs, precluding canonical RNA binding (Figure 4A). Nevertheless, the SETD1A RRM domain appears to allow RNA recognition in a non-canonical manner, involving a relatively broad surface (Figure 4A,B). These results are based on a series of the docking simulations with seven different RNA structures from randomized sequences (each 30–50 bp long), representing three-way junction, highly irregular RNA, K-turn, loop-turn, short triplex-containing, and quadruplex-like geometries (see Materials and Methods (Section 4) for details). The identified surface contains several positively charged amino acids (Figure 4B and 4D upper panel), such as Arg106, His128, Arg130, Arg132, and Lys133, known to be involved in RNA recognition [35]. Due to the lack of targeted structural mutational analysis, it is not possible to determine the exact mode of RNA binding and the relative involvement of specific amino acids. Nevertheless, this analysis corroborates the in vitro RNA binding results, underlining the physical capability of the SETD1A RRM domain to interact with RNAs.

Even though the RNA binding capacity of the RRM domain is evident, when considering its in vivo function, one must take into account that SET proteins act as parts of the large, multi-subunit COMPASS complexes. Apart from the obligatory complex components, different COMPASS complexes contain unique subunits that modulate their individual functions. WDR82 is a specific component of the SETD1A- and SETD1B- COMPASS complexes, while in yeast, a WDR82 homolog, Swd2, participates in the complex. The Swd2–Set1 interaction is mediated by a conserved region (CR) N-terminal to the first RRM in Set1 (Figure 4C,E). Importantly, the SETD1A–WDR82 interacting region mapped to a segment of SETD1A of similar position [22]. In order to gain a deeper insight into the interaction of WDR82 and the RRM domain of SETD1A and its possible influence on the RNA binding, we modeled the complex of these two proteins (Figure 4C,E) using AlphaFold2 multimer through Google Colaboratory Suite [36]. The resulting model of the confidently predicted complex between the N-terminus of human SETD1A (residues 15–200) and WDR82 (full length) shows that SETD1 proteins likely attach through two, extensive interaction sites onto the side of the WD40 β-propeller domain, while leaving the central, putative ligand binding surface untouched (Figure 4C). This ligand-binding site might be important for the targeting of the COMPASS complex onto the chromatin, as WDR82 is known to recognize the disordered tail of RNA polymerase II [23,24]. The model also reveals that the binding between WDR82 and SETD1A results in a fixed orientation of the subunits, where the positively charged, putative RNA binding interfaces of the SETD1A RRM are directly adjacent to two, similarly charged sides of WDR82 [37] (Figure 4E). Thus, it is possible that SETD1A and WDR82 have shared RNA partners and that specific RNA recognition is achieved by their combined surfaces. Superposition of the RNA-bound RRM models with the complex shows that the majority of simulated RNA strands do not clash with WDR82, but instead, the nucleic acid strands would continue towards the RNA-compatible interfaces of WDR82 (Figure 4D bottom panel).

### 2.4. SETD1A Localizes to Splicing Speckles in the Nucleus

**Figure 5:**
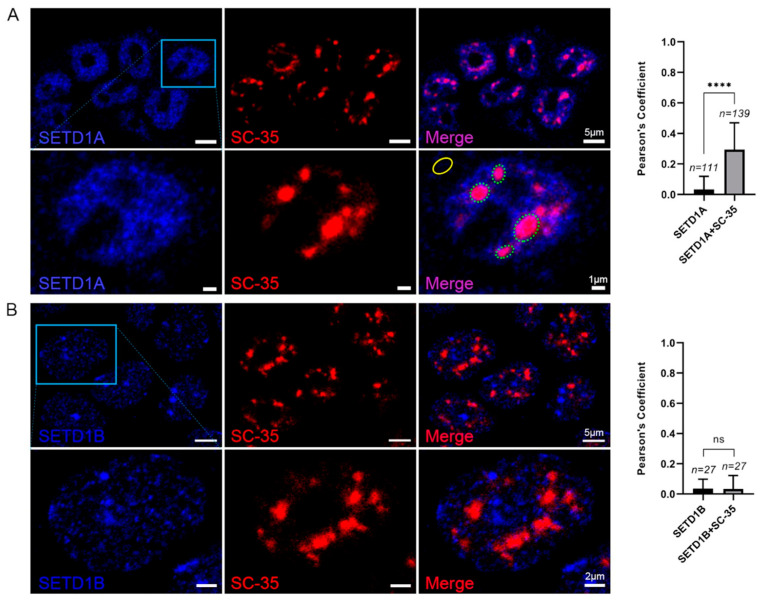
Colocalization of SETD1A or SETD1B with nuclear speckle marker, SC-35. Images represent the colocalization of SETD1A and the nuclear speckle marker, SC-35, in HEK293T cells. Dotted elliptical circles indicate the nuclear speckle and SETD1A condensates, and the solid elliptical circle shows the gated cytoplasmic SETD1A cluster that we considered as background for the colocalization (**A**). The same gatings were applied to select the regions of interest (ROIs) of SETD1B nuclear distribution and SC-35 (**B**). The graphs show the linear correlation of colocalization pixel intensity of SETD1A (**A**) or SETD1B (**B**) and SC-35 calculated with Pearson’s coefficient. The number of selected ROIs is shown as ‘*n*’ above the columns. Mean values and error bars are shown. Unpaired two-tailed Student’s *t*-test was applied for all statistical analysis, and *p* < 0.0001 is marked by ****.

The presence of splicing-related RNAs and the enrichment of functions related to RNA processing in the RNA interactome of the SETD1A indicate that, similar to yeast Set1, this protein may also have a role related to splicing. Since one of the major RNA processing bodies in the cells is the nuclear speckle [38], we performed colocalization experiments with SC-35 (Serine/arginine-rich splicing factor 2), a nuclear speckle marker protein [39]. At this stage, we aimed to compare the localization of SETD1A with that of SETD1B, as literature information indicated that these two proteins have non-overlapping staining [11] and we wanted to confirm that SETD1B does not localize to splicing speckles. The immunostaining of SETD1A showed a distinct, puncta-like distribution of the protein within the nucleus (Figure 5A) and a clear colocalization with SC-35, as Pearson’s correlation coefficient revealed a significant overlap between the pixel intensities of SETD1A and SC-35 (Figure 5A and upper bar graph, and Supplementary Figure S6). It was also evident that SETD1A can be found outside of the nuclear speckles as well, even appearing in a smaller amount in the cytoplasm (Figure 5A, bottom row), indicating a more diverse localization pattern of the protein than previously reported [11].

The localization of SETD1A to nuclear speckles was further confirmed by co-immunoprecipitation, where SC-35 could pull down SETD1A (Supplementary Figure S7), indicating that the two proteins directly interact. The intimate relationship of these proteins with the RNA maturation process is further corroborated by the presence of PSPC1 (Paraspeckle component 1) in the co-immunoprecipitation sample (Supplementary Figure S7), as paraspeckles are also considered to be involved in RNA processing [40].

SETD1B similarly showed puncta-like distribution and dominantly localized in the nucleus (Figure 5B), but it appeared to be excluded from the nuclear speckles, as there was no detectable colocalization with SC-35 (Figure 5B and bottom bar graph). This indicates that the two proteins have non-overlapping interaction partners that determine their intranuclear localization. It is important to note at this point that our attempts to find RNA interactors of SETD1B remained futile, as none of the six independent RIP experiments returned measurable amounts of RNA, suggesting that RNA binding may not be a significant feature of SETD1B.

Like SETD1A, SETD1B could also be found in the cytoplasm in a smaller abundance (Figure 5). While cytoplasmic localization is not considered the main form of occurrence of SETD1A and B proteins, it is not unprecedented and may even serve important functional purposes as observed in breast cancer [40].

## 3. Discussion

The notion that HKMTs bind and are regulated by different RNA species is not a new observation [41], but detailed information on their RNA interactome is relatively scarce, with mostly sporadic examples. Even though SETD1A and SETD1B contain RRM domains with published structures [34], neither the RNA recognition of the separated domains, nor that of the full-length proteins, have been characterized.

Our RIP-Seq results unequivocally prove that SETD1A possesses an extensive RNA binding capacity involving both coding and non-coding RNAs, while SETD1B has a less efficient RNA interaction capability.

RNA binding can have several different roles that may influence either the protein or the RNA species in the interaction.

mRNA stability and processing can be regulated by the interacting proteins, which influences their life cycle and the availability of the proteins they encode. As SETD1A appears to bind the mRNAs of several histone modifier enzymes, it is possible that it plays a role in the regulation of histone modification independently of its methyltransferase activity, through controlling the mRNA pool of these proteins. A similar regulatory activity has been described for the yeast Set1, which has been shown to bind its own mRNA [42], in an at least partially cotranslational manner. The fact that the identified mRNAs rarely contain intronic regions in our dataset indicate the possible existence of a similar binding pattern and activity for SETD1A.

One of the most abundantly studied roles of RNA binding by HKMTs is the specific targeting of the histone modifier to its cognate genomic loci by interacting with long non-coding RNAs. Several pieces of compelling evidence support this model in the case of EZH2, the methyltransferase component of the Polycomb Repressive Complex 2 (PRC2) [43,44,45]. Even though our experiments did not aim to find similar regulatory interactions, several of the lncRNAs identified in our RIP-Seq experiments, such as TERC [46] and HOTAIR [47], are known to act in a similar manner. Further, targeted experiments will be needed to clarify such roles of the lncRNA partners of SETD1A.

The interaction of SETD1A with splicing-related non-coding RNAs raises the possibility of its involvement in the regulation of RNA processing. This possibility is further corroborated by the colocalization of SETD1A to nuclear speckles (also known as splicing speckles) and its direct interaction with the splicing factor SC-35. In fact, the localization of SETD1A to nuclear speckles has been already mentioned in the literature, but only to indicate that the protein appears in a speckle-like pattern within the nucleus [11], and no details of the specific organelle have been provided. Our results clarify that out of the several different membraneless organelles in the nucleus that appear in distinct, puncta-like condensates, it is the splicing speckle that contains SETD1A. The exact role of SETD1A in these speckles remains to be determined. However, given that RNA binding does not appear to be predominantly cotranscriptional, it is possible that SETD1A has an indirect function in the regulation of splicing. One plausible role would be the methylation of splicing factors, similarly to SETMAR, which has been shown to methylate snRNP70 [48]. Lysine methylation of splicing factors is known to influence their activity through the regulation of their interactions, as in the case of RBM25, where the K77me1 modification blocks the interaction with SRSF2 [49]. Moreover, lysine methylation appears to be a widespread phenomenon among splicing-related proteins [50], although the methyltransferases responsible for the modification remain largely elusive. Positioning SETD1A in splicing speckles through either the interaction with its component proteins or with the constituent RNAs would present an ideal situation for the execution of such methylation events.

Our finding that PSPC1, a component of paraspeckle could also be pulled down with SC-35, indicates that these organelles are in dynamic contact, and probably exchange components. The unmistakable difference in the subnuclear localization of SETD1A and SETD1B has been attributed to the specific differences within the two methyltransferases [11] and our results that show a limited RNA interacting capacity for SETD1B offer a plausible explanation for that. If the interaction with RNAs is necessary to target SETD1A to splicing speckles, the lack of strong RNA binding in SETD1B could lead to the exclusion of this protein from the same organelle.

While we have observed several similarities in the RNA binding of SETD1A and yeast Set1, an important difference between these two methyltransferases is that the RRM domain of Set1 cannot bind RNA on its own [19], but the RRM of SETD1A is an active RNA binding unit, showing strong affinity and limited specificity towards the protein’s native RNA partners. Structural analysis of the crystal structures and in silico docking performed with the SETD1A RRM domain reveals that the RNA interaction probably occurs through a broad, putative, non-canonical RNA binding region and is not mediated by the RNA recognition surface of the well-characterized RRM domains in the literature. The profound differences in the surface of RRM domains might explain why the yeast Set1 RRM1 binds RNA only when paired with the more positively charged RRM2.

Seeing these differences and that the predicted RNA interacting surfaces of the human SETD1A are evolutionarily not strictly conserved (the entire predicted surface is different in SET1B, and also varies considerably across animal species), we argue that RNA binding may not be a fully conservative feature in the function of the SET1 protein family. In the instances it occurs, the molecular mechanism of the interaction has been continuously changing throughout the evolution of these proteins.

## 4. Materials and Methods

### 4.1. Experimental Models

All in-cell experiments in this study were performed on human embryonic kidney (HEK293T) cells. The cell line was a kind gift from the laboratory of Gergely Szakács (Drug Resistance Research Group, Research Centre for Natural Sciences, Budapest H-1117, Hungary). Cell Line Authentication performed by Eurofins Genomics Europe Shared Services GmbH (85560 Ebersberg, Germany) confirmed 100% authenticity of the cells.

### 4.2. Cell Culture Conditions

HEK293T cells were grown in high glucose Dulbecco’s Modified Eagle Medium (DMEM) (Capricorn Scientific GmbH, Ebsdorfergrund, Germany, cat#: DMEM-HA) supplemented with 1× Penicillin-Streptomycin (Thermo Fisher Scientific, Waltham, MA, USA, cat#: 15070063), and 10% Fetal Bovine Serum (Merck KGaA, Darmstadt, Germany, cat#: F9665-500ML) in a humidified environment at 37 °C and 5% CO_2_. Cells were passaged regularly every 48 h at 2 × 10^6^ cells per 10 cm cell culture dish (AVANTOR, Radnor, PA, USA, #734-2817) up to 10 passages.

### 4.3. Nuclear Extraction for RNA Immunoprecipitation

One 10 cm cell plate of HEK293T cells at 80–90% confluency was used for 4 immunoprecipitation (IP) reactions. Attached cells were washed twice with ice-cold phosphate buffered saline (PBS), then UV crosslinked once at 150 mJ/cm^2^ on ice using CL-1000 UV crosslinker. Crosslinked cells were collected by scraping in 5 mL of ice-cold PBS, and spun at 1000 rpm for 2 min. Pelleted cells were lysed in 100 µL/1 × 10^6^ cells of cytoplasmic extraction buffer (20 mM Tris-HCl pH 7.4, 10 mM NaCl, 3 mM MgCl_2_, 0.5 mM DTT, 0.05% NP-40, 1× protease inhibitor cocktail (Merck KGaA, Darmstadt, Germany, cat#: S8820)) for 15 min on ice, then centrifuged at 4000 rpm for 5 min at 4 °C. To get rid of cytoplasmic contaminants, the nuclear pellet was washed once with 1 mL of ice-cold PBS. Nuclei were lysed in 200 µL/per 10 × 10^6^ cells of nuclear extraction buffer (50 mM Tris-HCl pH 7.4, 150 mM NaCl, 1% NP-40, 5 mM EDTA, 1 mM EGTA, 1 mM PMSF, 50 mM NaF, 1× protease inhibitor cocktail, 4 U/mL RNase inhibitor (Merck KGaA, Darmstadt, Germany, cat#: 3335402001)) for 30 min on ice with frequent vortexing. Lysed nuclei were centrifuged at 16,000× *g* for 10 min at 4 °C. Nuclear proteins in supernatant were transferred into a new ice-chilled 1.5 mL microfuge tube and diluted with 800 µL of dilution buffer (nuclease-free PBS pH 7.4 and 6.5% glycerol). The diluted nuclear lysate was pre-cleared with 60 µL protein A/G coated slurry magnetic beads (Thermo Fisher Scientific, Waltham, MA, USA, cat#: 88802) for 2 h at 4 °C on end-over-end rotation. Beads were removed on a magnetic rack and the cleaned lysate was transferred to a new 1.5 mL microfuge tube for fresh RNA immunoprecipitation.

### 4.4. RNA Immunoprecipitation (RIP)

One-quarter of the above precleaned nuclear extract with 6 µg of either anti-SETD1A or RIP negative control IgG from the same species (see the list of antibodies used in the Key Resources Table S5) was incubated overnight at 4 °C on end-over-end rotation, and 25 µL of the same lysate was kept as 10% input at −80 °C for RIP-qPCR. On the next day, 12 µL of protein A/G coated slurry magnetic beads (Thermo Fisher Scientific, Waltham, MA, USA, cat#: 88802) per IP were washed 3 times with 200 µL of bead-washing buffer (nuclease-free PBS pH 7.4, 0.5% Tween-20). Nuclear protein complex-antibody complexes were incubated with washed beads for 2 h on end-over-end slow rotation at 4 °C. Ribonucleoprotein (RNP)-bound magnetic beads were briefly spun at 3000 rpm and separated on a magnetic rack for 5 min at 4 °C. The supernatants were discarded and beads were washed 2 times with 200 µL of low-salt wash buffer (150 mM NaCl, 20 mM Tris-HCl pH 8, 2 mM EDTA, 1% Triton X-100, 1× protease inhibitor cocktail (Merck KGaA, Darmstadt, Germany, cat#: S8820) and 80 U/mL Protector RNase Inhibitor (Merck KGaA, Darmstadt, Germany, cat#: 3335402001)). To avoid the elution of non-specific protein complexes bound to the microfuge tube wall, beads were transferred to new pre-chilled microfuge tubes, and washed again 2 times with high-salt wash buffer (500 mM NaCl, 20 mM Tris-HCl pH 8, 2 mM EDTA, 1% Triton X-100, 1× protease inhibitor cocktail, 80 U/mL Protector RNase Inhibitor) and 1 more time with TE buffer (15 mM Tris-HCl pH7.4, 5 mM EDTA). Bound RNAs were eluted with 200 µL of IP elution buffer (100 mM Tris pH 8, 10 mM EDTA, 1% SDS, 0.2 µg/µL Proteinase K) and 10% input samples were brought up to a final volume of 200 µL with the same elution buffer. The elution was performed at 60 °C for 30 min with frequent vortexing, then cooled down on ice for 5 min. After a brief spinning at 3000 rpm, the eluates of RIP reactions were transferred to new microfuge tubes and proceeded to RNA purification.

### 4.5. RNA Purification

Immunoprecipitated RNAs with their corresponding 10% inputs were extracted according to Imprint^®^ RNA Immunoprecipitation protocol (Merck KgaA, Darmstadt, Germany, cat#: RIP) or following the manufacturer’s instructions of Direct-Zol RNA Miniprep kit (Zymo Research, Irvine, CA, USA, cat#: R2050). Depending on the downstream applications, purified RIP RNAs were either pelleted for next generation sequencing or re-suspended/eluted in 20 µL of nuclease-free water for cDNA synthesis followed by RIP-qPCR.

### 4.6. RNA Sequencing Data Analysis

We prepared independent biological replicates for RNAs immunoprecipitated with anti-SETD1A (three replicates), anti-SETD1B (six replicates), or a negative control antibody from the same species (Key Resources Table S5). SETD1B trials did not return sufficient RNA amounts for sequencing. After rRNA depletion, 1 µg of RNA of each sample was used for RIP-sequencing protocol on an Illumina NovaSeq 6000 high throughput NGS platform (Novogene, Cambridge, UK).

NGS results were analyzed as follows. After trimming the adaptors and the low quality sequences with the Trimmomatic tool [51], the obtained 150 bp paired-end reads were aligned to the GRCh38 human transcriptome using HISAT2 [52]. Aligned reads were manipulated (converted to BAM format, sorted, and indexed) using Samtools [53]. Transcriptome assembly and differential expression analysis were performed using the Cufflinks program package (Cufflinks, Cuffmerge, Cuffquant, Cuffdiff) [54]. Differences between the identified transcripts were calculated based on the differences in FPKM (fragments per kilobase of transcript per million mapped fragments) numbers of the SETD1A and the negative control samples. We considered a gene as a positive hit if it had a higher FPKM value in the RIP sample than in the negative control.

### 4.7. Bioinformatics Analyses

Panther protein class analysis was performed using the PANTHER Classification System online tool [55,56]. For visual exploration of genomic data, Integrative Genomics Viewer (IGV) was used [57].

### 4.8. cDNA Synthesis

For each cDNA synthesis reaction, equal volumes of RIP RNAs and their corresponding 10% input were reverse transcribed according to SuperScript™ III Reverse Transcriptase kit (Thermo Fisher Scientific, Waltham, MA, USA, cat#: 18080044) manufacturer’s instructions. Reactions were briefly vortexed and spun down for 5 s. PCR cycling conditions were applied as follows: incubation at 25 °C for 5 min followed by 50 min incubation at 50 °C, then 10 min at 55 °C. Finally, the reactions were heat inactivated at 70 °C for 15 min.

### 4.9. RT-qPCR

RT-qPCR reaction mixes were prepared as follows: 10 µL TaqMan™ Fast Advanced Master Mix (Thermo Fisher Scientific, Waltham, MA, USA, cat#: 4444557), 1 µL TaqMan probe (Key Resources Table S6), 7 µL nuclease-free water. The reaction mixture at 18 µL/well was loaded into corresponding wells of a 96-well RT-qPCR plate, (Thermo Fisher Scientific, Waltham, MA, USA, cat#: 4346907 or cat#: N8010560), and 2 µL of cDNA was added per reaction. Using QuantStudio^TM^ 6 pro Real Time-qPCR (Thermo Fisher Scientific, Waltham, MA, USA,) machines, fast comparative amplification cycling setup was applied for 40 cycles. To calculate the yield (% input) and specificity (fold enrichment) of each probe in specific RIP reactions compared to negative control RIP, cycle threshold values were analyzed to obtain ∆∆Ct according to the method published by Marmisolle et al. [58].

### 4.10. SETD1A_RRM Expression and Purification

DNA sequence coding for SETD1A_RRM (89-197, UniProt: O15047) domain in pET28-MHL vector was a gift from Cheryl Arrowsmith (Addgene plasmid #32868; http://n2t.net/addgene:32868; accessed on 12 April 2021; RRID: Addgene_32868). The construct was transformed into competent *E. coli Rosetta* cells and grown in LB medium (Merck KgaA, Darmstadt, Germany, cat#: L3022) containing 0.05 mg/mL kanamycin sulfate (Thermo Fisher Scientific, Waltham, MA, USA, cat#: 11815024) overnight at 37 °C with shaking at 180 rpm. After inoculation with the starter cell culture into fresh LB medium containing 0.05 mg/mL kanamycin, the cells were grown to OD_600_ = 0.6. Induction was performed for 4 h at 30 °C by 0.5 mM IPTG, then cells were pelleted by centrifugation (4000 rpm, 20 min, 4 °C) and incubated at room temperature for 30 min in lysis buffer (20 mM Tris, 200 mM NaCl, 20 mM imidazole, 1 mg/mL Lysozyme, 50 U/mL Universal Nuclease for Cell Lysis (Thermo Fisher Scientific, Waltham, MA, USA, cat#: 88700) pH 7.5, and EDTA-free SIGMA*FAST* Protease Inhibitor Cocktail Tablets (Merck KgaA, Darmstadt, Germany, cat#: S8830)) with vigorous shaking. After sonication the cell debris was removed by centrifugation (12,100 rpm, 40 min, 4 °C). The supernatant was filtered through a 0.2 μm nitrocellulose membrane then purified over a HisTrap HP column on an AKTA Explorer system using a gradient elution of two buffers (Buffer A: 20 mM imidazole, 200 mM NaCl, 20 mM Tris. pH 7.5, Buffer B: 1 M imidazole, 200 mM NaCl, 20 mM Tris, pH 7.5). Elution fractions containing sufficiently pure proteins were transferred to distilled water via overnight dialysis. After centrifugation (12,100 rpm, 40 min, 4 °C) the supernatant was lyophilized and stored at −20 °C until usage. A representative purification result is shown in Supplementary Figure S8.

### 4.11. Far-UV Circular Dichroism

CD measurements were performed on a Jasco J-810 (Jasco, Tokyo, Japan) spectropolarimeter in 0.1 mm quartz cells, in DEPC-treated assay buffer (50 mM Tris pH: 7.5, 150 mM KCl, 2,5 mM MgCl_2_, 0.05% NP-40, 1 mM DTT). Far-UV CD spectra were recorded in the 180–260 nm wavelength range with a scanning speed of 50 nm/min, 1 nm bandwidth, and 2 s data integration time. Ten scans were accumulated at room temperature. CD spectra were quantitatively analyzed for the secondary structure composition using the BeStSel method [31,32] (http://bestsel.elte.hu), accessed on 20 May 2023.

### 4.12. RNA In Vitro Transcription, Purification, and Labeling

HOTAIR440, SNHG8, TP53TG1, NEAT1_2, SCARNA7, and AGAP2_AS1 DNA sequences cloned into pEX-A128 vector with a T7 promoter sequence at the beginning and an EcoRV restriction site at the end were purchased from Eurofins Genomics (Ebersberg, Germany). The coding sequence for SETD1B_RRM domain (85–200 aa) was purchased from Eurofins Genomics and subcloned into pET22b vector at BamHI and XhoI sites. The coding sequence for the N-terminal half of SETD1A (SETD1A_N, 1–836 aa) was cloned into a pET22b vector with a SalI restriction site at the end. After 3 h digestion with EcoRV, XhoI, or SalI restriction enzymes at 37 °C, respectively, the gel-purified, linearized DNA template was used to synthesize RNA by in vitro transcription carried out with New England BioLabs HiScribe™ T7 Quick High Yield RNA Synthesis Kit (NEB, cat#: E2050S).

Random 50 nucleotide RNA was an artificial randomized RNA sequence. The following primers were used as a template during the in vitro transcription:

T7 promoter region followed by 50 nt forward oligo:

AAGAATGGCCTCGCGGAGGCATGCGTCATGCTAGCGTGCGGGGTACTCTT and

50 nt reverse oligo:

AAGAGTACCCCGCACGCTAGCATGACGCATGCCTCCGCGAGGCCATTCTTCTATAGTGAGTCGTATTA

Transcribed RNA:

AAGAAUGGCCUCGCGGAGGCAUGCGUCAUGCUAGCGUGCGGGGUACUCUU

For MST measurements, the in vitro transcription was carried out in the presence of Amynoallyl-UTP-X-Cy5 (Jena Bioscience, Jena, Thuringen, Germany, cat#: NU-821-X-CY5-S).

After transcription, the remaining DNA template was eliminated with DNaseI treatment. The transcribed RNA was purified using Macherey-Nagel NucleoSpin^®^ RNA Clean-up XS Kit (cat#: 740903.50).

Biotinylation of the RNAs for the EMSA measurements was performed using a Pierce™ RNA 3′ End Biotinylation Kit (Thermo Fisher Scientific, Waltham, MA, USA, cat#:20160) according to the instructions of the manufacturer. Overnight incubation at 16 °C was applied for the ligation of the biotin label.

The quality and intactness of the labeled or unlabeled purified RNA was analyzed by native and formaldehyde agarose gel electrophoresis. Final RNA concentration was determined using an Implen NanoPhotometer™ N60 Spectrophotometer (Münich, Germany). Purified RNA was stored at −80 °C until usage in the presence of Roche Protector RNase Inhibitor (Merck KgaA, Darmstadt, Germany, cat#: 3335399001). Before usage the RNA sample was refolded by incubation at 75 °C for 5 min and then allowed to cool down to room temperature.

### 4.13. Electrophoretic Mobility Shift Assay (EMSA)

LightShift^®^ Chemiluminescent RNA EMSA Kit (Thermo Fisher Scientific, Waltham, MA, USA, cat#: 20158) was used for the EMSA experiments. Binding, electrophoresis, and detection of the tested RNAs with the proteins were carried out following the protocol of the kit. Briefly, proteins of varying concentrations were incubated with 2 nM of biotinylated RNAs for 30 min at room temperature, then loaded on 6% native polyacrylamide gels and ran at 100 V in 0.5× TBE buffer for varying durations depending on the size of the RNA. RNAs were transferred to nitrocellulose membranes using a Trans-Blot^®^ Turbo™ Transfer System (Bio-Rad, Hercules, CA, USA) and crosslinked to the membrane by UV-light. After washing and blocking, biotin-labeled RNA was detected by chemiluminescence using Streptavidin-Horseradish Peroxidase Conjugate (provided as part of the kit).

### 4.14. Microscale Thermophoresis (MST)

RNA-protein binding assays were carried out on a microscale thermophoresis system (Monolith NT. 115 from NanoTemper Technologies, München, Germany). Standard-treated Monolith capillaries (NanoTemper, cat#: MO-K002) were used for the measurements. Instrument settings are presented in Table 2.

RNA concentrations were set to give an initial raw fluorescence between 300 and 500 counts and varied between 3 and 25 nM. All experiments were performed at room temperature in DEPC-treated assay buffer (50 mM Tris pH: 7.5, 150 mM KCl, 2.5 mM MgCl_2_, 0.05% NP-40, 1 mM DTT).

Normalized fluorescence values after 1.25 s after turning on the IR laser were used as T-jump values.

### 4.15. In Silico Structural Modeling

To analyze the potential RNA binding surfaces of SETD1A and Set1 RRM domains, we used both experimentally determined crystal structures (PDB: 3S8S, 8ILY, 2J8A) [19,34] as well as AlphaFold2-generated models downloaded from the EBI web server (https://alphafold.ebi.ac.uk/, accessed on 6 April 2023) [59]. The SETD1A-WDR82 dimer was modeled using ColabFold v1.5.2 [35] with default settings (no relaxation), using the sequence of human SETD1A N-terminus (UniProt accession O15047, residues 15–200) and human WDR82 (UniProt: Q6UXN9, full length). All output structures were directly superimposable, with minimal variability.

To gain some insight into the RNA binding capacity of the RRM domain, in addition to R50 (Supplementary Figure S9), we generated 6 further, randomized RNA sequences (50 bp each), picking those that reflected essentially different 3D structures according to RNAcomposer [60,61] outputs. The modeled structures were trimmed (to 30–50 long cores), removing loose, uncoordinated terminal RNA stretches at 3′ and 5′ ends. The resulting compact RNA structures were subsequently docked to the SETD1A RRM domain (PDB: 8ILY) using HDOCK [62,63,64]. The 10 best structures from each docking session were then filtered, removing models where the protein bound too close to RNA ends (within 5 angstroms to a terminal nucleotide). We also removed all structures where the RNA clearly clashed with polypeptide chains in the larger, modeled SETD1A-WDR82 complex, arguing that such ribonucleotide binding to the RRM domain would be non-physiological.

The remaining 36 perfectly compatible structures (out of the initial 70) were then used to evaluate RNA-binding surfaces on the RRM domain (using a distance < 5 angstroms at any atoms of a residue for a potential contact). Selected docked structures are also shown in Figure 4A,D.

Structural figures were prepared using PyMol (v. 1.8, Schrödinger Inc., New York, NY, USA)

### 4.16. Co-Immunoprecipitation (Co-IP)

Co-IPs were performed the same way as nuclear extractions and RIPs with some modifications. About 3 × 10^7^ cells/co-IP were crosslinked with 1 mL of 0.75% formaldehyde for 8 min at RT and quenched with 125 mM of glycine for 5 min at RT, then 10 min on ice. Cells were lysed in 1 mL per 10 × 10^6^ cells of cytoplasmic extraction buffer. The nuclear pellets were resuspended in 750 µL per 3 × 10^7^ cells of nuclear extraction buffer without RNase inhibitor for 15 min on ice with frequent vortexing, then diluted with an equal volume of dilution buffer (PBS pH 7.4, 10% glycerol). The nuclei were sonicated at low amplitude (level 4) for 6 cycles (6 s pulse, 6 s rest) using Diagenode SA BIORUPTOR Plus in 200 µL/0.5 mL thin-wall transparent microfuge tubes (Axygen PCR-05-C-500 0.5 mL flat cap). Nuclear lysates were centrifuged at 16,000× *g* for 10 min at 4 °C. Supernatants were mixed and equal volumes corresponding to 3 × 10^7^ nuclei (≃1 mg of nuclear protein) were used per each co-IP. About 60 µg of total cell extract or nuclear lysate in a final volume of 25 µL was kept at −80 °C for the co-IP input. Co-IP reactions were pre-cleaned with 30 µL protein A/G coated magnetic beads (Thermo Fisher Scientific, Waltham, MA, USA, cat#: 88802) for 1 h at RT on end-over-end rotation, then the beads were discarded. For each IP reaction, 6 µg of anti-target or negative control IgG of the same species was used (see the list of antibodies used in the Key Resources Table S5) with overnight incubation at 4 °C on end-over-end rotation. On the next day, the nuclear lysate-antibody complex was incubated with 30 µL protein A/G coated magnetic beads for 1 h at RT on end-over-end rotation. After this, the beads were successively washed 2 times with a low-salt wash buffer, 2 times with a high-salt wash buffer, and 1 time with a TE buffer. Protein complexes were eluted with 25 µL of 1× Laemmli buffer containing 25 mM DTT, and input samples were mixed with 7 µL of 4× Laemmli buffer containing 100 mM DTT. All samples were denatured at 95 °C for 10 min. After cooling down at RT for about 15 min, magnetic beads were pulled aside on a magnetic rack and the supernatants were transferred to Western blotting.

### 4.17. Western Blot

Proteins were separated on 1 mm thick 4–20% gradient Mini-PROTEAN^®^ TGX™ Precast Protein Gel gels (Bio-Rad, cat# 4561094). A quantity of 6 µL of protein ladder (Thermo Fisher Scientific, Waltham, MA, USA, cat#: 26619) was used per lane. Proteins were separated at 180 volts for 1 h and transferred onto PVDF membranes (Bio-Rad, cat#: 10026933) according to instructions of the Trans-Blot Turbo Transfer System manual (Bio-Rad, cat#: 1704150), using 1× turbo transfer buffer (Bio-Rad, Hercules, CA, USA, cat#: 10026938). After transfer, membranes were washed twice with tris buffered saline containing 0.1% Tween-20 (TBS-T), then blocked for 1 h at RT in 5% non-fat skim milk powder dissolved in TBS-T. Blocked membranes were washed twice in TBS-T, and probed overnight at 4 °C with primary antibodies (Key Resources Table S5) diluted in TBS-T. Then, unbound antibodies were washed out 3 times with 15 mL of TBST, each for 10 min on shaking at RT. Washed membranes were blotted for 1 h at RT with horseradish peroxidase-conjugated secondary antibodies (Key Resources Table S5) diluted in TBST. Unbound antibodies were washed out 3 times with 15 mL of TBS-T, each for 10 min on shaking at RT. Finally, membranes were subjected to chemiluminescence revealing using a Bio-Rad ChemiDoc MP imaging system.

### 4.18. Immunoflourescence Staining (IFS)

For immunofluorescence assays, cells were seeded on pre-cleaned and sterilized 12 mm round coverslips at 3 × 10^4^ cell density per well in 24-well plates. At the desired confluency, cells were fixed with 4% paraformaldehyde/PBS for 10 min at RT. After 3 subsequent washes with 0.1% Tween-20/PBS (PBS-T), each for 5 min, cells were permeabilized with 0.5% Triton X-100/PBS for 10 min at RT, then blocked with blocking solution (2% BSA, 5% fetal bovine serum, 0.2% porcine skin gelatin, 0.1% Triton X-100 in PBS) for 1 h at RT, followed by incubation overnight at 4 °C with primary antibodies (see the list of antibodies used in the Key Resources Table S5) diluted in the blocking solution. Primary antibodies were washed out 4–6 times with PBS-T, and cells were incubated for 1 h at RT with fluorophore-conjugated secondary antibodies (see the list of antibodies used in the Key Resources Table S5) diluted in the blocking solution. After 4 successive washes with PBS-T and 2 washes with sterile distilled water, coverslips were mounted on microscopic slides using Fluoromount-G (Thermo Fisher Scientific, Waltham, MA, USA, cat#: 00-4958-02) and kept at 4 °C until IFS detection with a confocal microscope.

### 4.19. Confocal Microscopy

Colocalization was assessed via an LSM 710 inverted confocal microscope (Carl Zeiss, Oberkochen, Germany) with a Plan-Apochromat 63×/1.40 Oil DIC M27 objective. Images were recorded by the ZEN 3.8 software (Carl Zeiss, Oberkochen, Germany) using constant image acquisition settings. Images were acquired from random fields and randomly selected replicates. All images were processed with Carl Zeiss ZEN 2012 Blue Edition 3.8 software.

### 4.20. Co-Localization Intensity Analysis

Images were analyzed with an enhanced version of ImageJ called Fiji. ImageJ bundled with 64-bit Java 1.8.0_172 was downloaded from https://imagej.nih.gov/ij/download.html, accessed on 1 December 2022, and BIOP JACoP, a Fiji plugin for co-localization intensity analysis, was downloaded from https://biop.epfl.ch/Fiji-Update/ accessed on 1 December 2022. This plugin, depending on the space methods of Pearson, Manders, Li, and more, implements the pixel intensity correlation. After background subtraction, images with two channels were analyzed based on Pearson’s correlation, which measures the strength of the linear correlation between the pixel intensity of the two channels ranging from −1 to +1. Here, the value of −1 means complete artifact co-localization, 0 is no co-localization, and +1 is complete co-localization.

### 4.21. Statistical Analysis

#### 4.21.1. qPCR Experiments

Experiments were carried out on at least three independent biological replicates and the fold enrichment of immunoprecipitated RNAs was calculated as 2^(−ΔΔCt [SETD1A RIP/NS Rb IgG RIP])^. Statistical analysis was performed using GraphPad Prism 8.0.1, one-way ANOVA.

#### 4.21.2. Colocalization Experiments

All experiments were carried out on at least three independent biological replicates and distinct samples were selected for relative measurements. For quantification of colocalization intensity, all images were taken randomly from the same sample, and relative quantifications were reproduced from independent biological replicates to recover similar results. The nuclear speckle protein condensates or cytoplasmic SETD1A clusters were gated as regions of interest (ROIs) for the measurement of Pearson’s coefficient. Using GraphPad Prism 8 (GraphPad Software, Boston, MA, USA), statistical *p* values were calculated by unpaired two-tailed Student’s *t*-test, and *p* < 0.05 to *p* < 0.0001 were marked by * to ****, respectively.

## 5. Conclusions

Our presented results show for the first time that SETD1A is capable of interacting with several RNA molecules in the cells. We reproductively showed that the isolated RRM domain is capable of RNA binding on its own, in contrast with the yeast Set1 RRM. We also provided structural analysis of the possible RNA binding surfaces on the SETD1A RRM domain and identified the main differences between these regions in SETD1A and Set1, explaining the inability of the latter to interact with RNAs on its own.

Nevertheless, our studies also have some limitations that need to be taken into consideration and investigated further. One such shortcoming is the lack of information on the essentiality of the RRM domain for RNA binding within the protein. It is becoming more widely acknowledged that non-canonical RNA recognition elements abound in protein sequences [65,66], and other HKMTs have already been shown to bind RNAs in the absence of an RRM domain [67]. Moreover, yeast Set1 has been proven to be able to bind RNAs independently on the RRM [68]. Our results indicate that the separated RRM domain of SETD1A possesses a limited target specificity, indicating the possible existence of other regions within the protein that fine-tune RNA recognition. Whether these are capable of RNA binding on their own or are involved in the determination of specificity [69] needs to be determined through further, targeted studies.

Another limitation is our inability to conclusively show the RNA binding capacity of SETD1B. Although our experiments consistently indicate a lack of robust RNA binding activity, we cannot conclude unequivocally that the RNA binding capability is completely missing in the case of SETD1B.

## Figures and Tables

**Table 1:** Binding affinities between SETD1A RRM and different RNAs determined by MST. Values not calculated by the evaluation software are indicated as not determined (n.d.).

RNA	K_d_ T-Jump (μM)	K_d_ Thermophoresis (μM)	K_d_ Back Diffusion (μM)
SNHG8 (570 nt)	0.87	2.19	1.19
TP53TG1 (707 nt)	0.67	0.69	1.05
HOTAIR440 (440 nt)	2.09	1.39	1.45
NEAT1_2 (2976 nt)	1.11	1.52	1.89
AGAP2_AS1 (1567 nt)	n.d.	n.d.	n.d.
SCARNA7 (330 nt)	1.28	0.31	1.17
SETD1B (511 nt)	0.31	n.d.	0,31
SETD1A (2643 nt)	0.21	n.d.	0.06
Random50 (50 nt)	15.50	28.14	11.77

**Table 2:** Instrument settings for the MST measurements.

LED Power (%)	MST Power (%)	Before MST (s)	MST on (s)	After MST (s)	Delay (s)
20–40	40	5	30	5	25

## Data Availability

The datasets generated and analyzed during the current study are available from the corresponding author on request.

## Supplementary Materials

The supporting information can be downloaded at: https://www.mdpi.com/article/10.3390/ijms242216032/s1.

## Appendix A

Sequences of the RNAs used in the in vitro binding studies.

**Small Cajal body-specific RNA 7 (SCARNA7), 330 nt**

**AGAP2 antisense RNA 1 (AGAP2-AS1), 1567 nt**

**Nuclear Paraspeckle Assembly Transcript 1_2 (12_13k segment), NEAT1_2, 976 nt**

**HOX transcript antisense RNA (1-440), HOTAIR440, 440nt**

**TP53 target long non-coding RNA, TP53TG1, 707 nt**

**Small nucleolar RNA host gene 8, SNHG8, 570 nt**

**Random 50 nucleotide, 50 nt**

**SETD1B, 511 nt**

**SETD1A, 2643 nt**
