# Supplementary material for: In Vivo and In Vitro Characterization of the RNA Binding Capacity of SETD1A (KMT2F)
Source: Int J Mol Sci. 2023 Nov 7;24(22):16032. doi: 10.3390/ijms242216032 (PMC10671326; doi:10.3390/ijms242216032)
Supplement: Supplementary file 1 [file ijms-24-16032-s001.zip › Supplementary Figures_updated.pptx]

## Slide 1
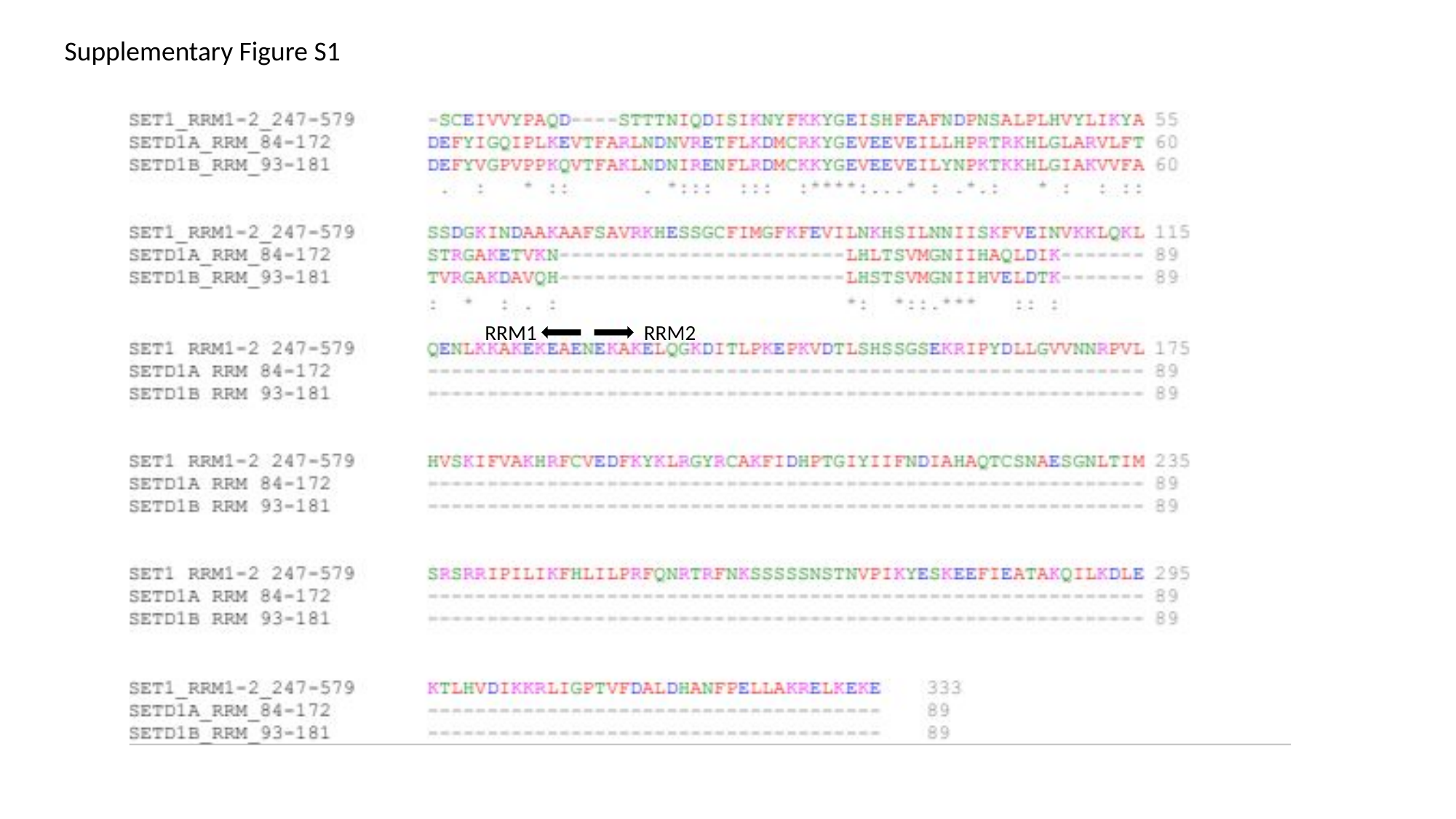

Supplementary Figure S1
RRM1
RRM2

## Slide 2
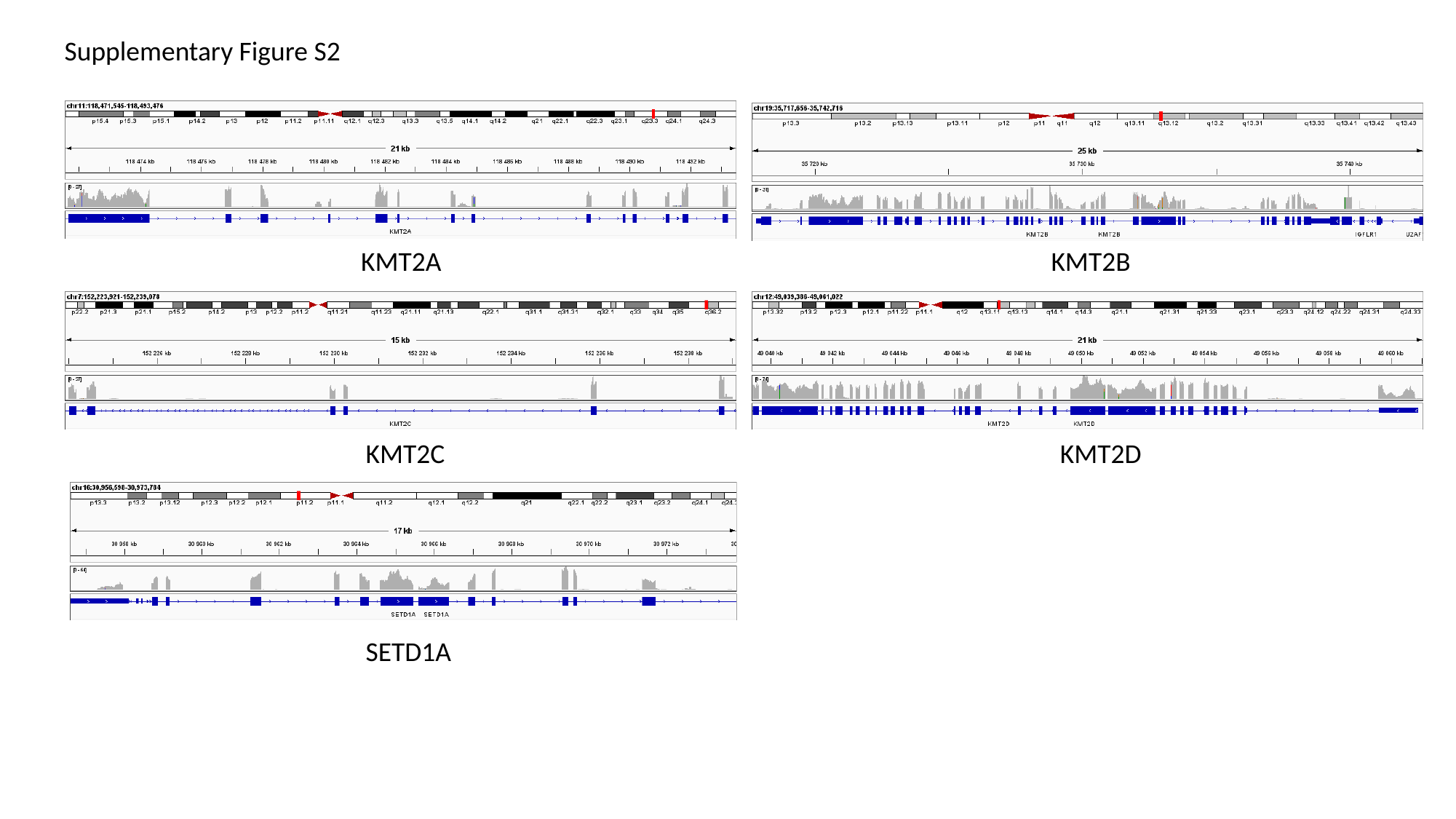

Supplementary Figure S2
KMT2A
KMT2B
KMT2C
KMT2D
SETD1A

## Slide 3
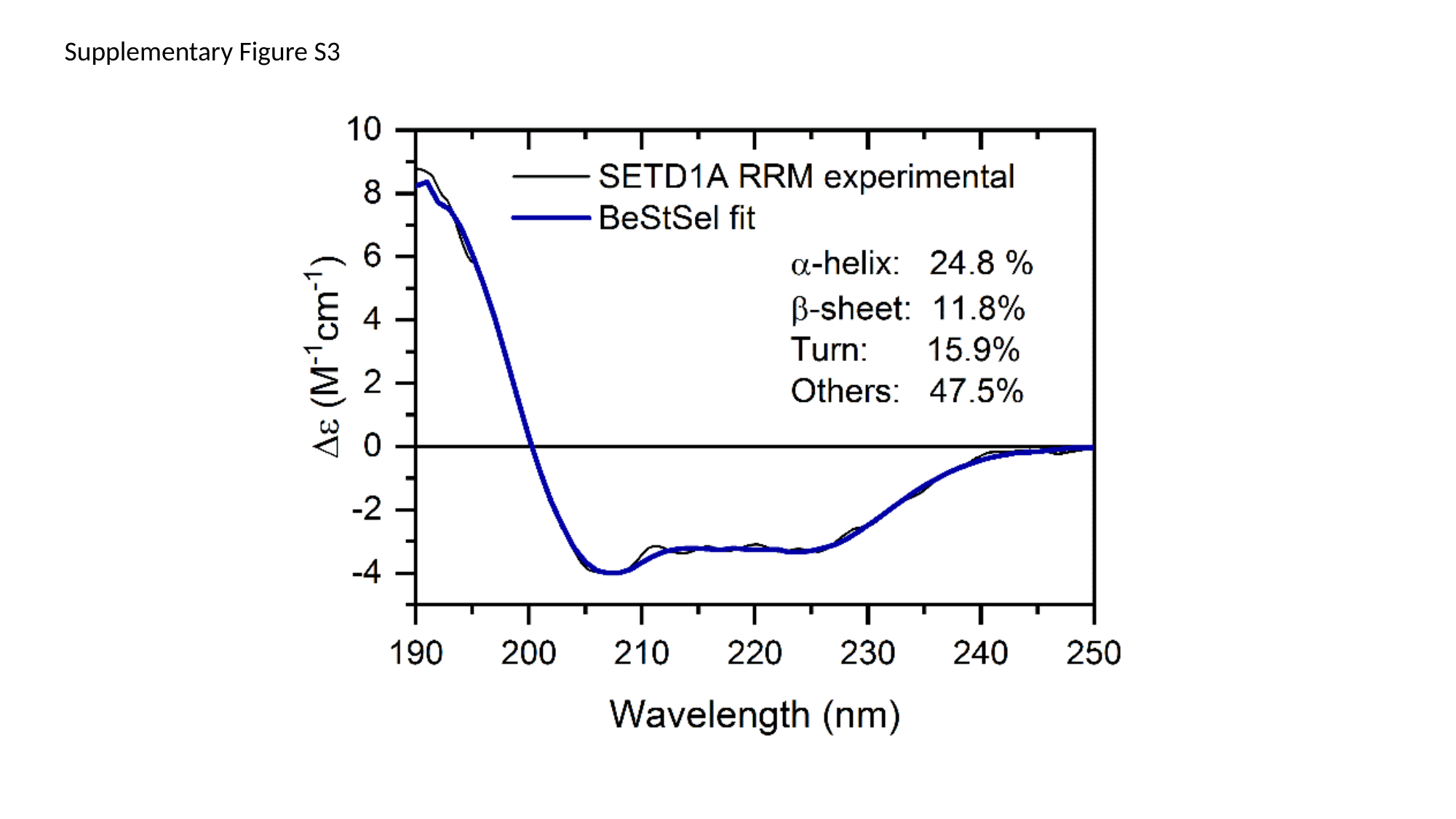

Supplementary Figure S3

## Slide 4
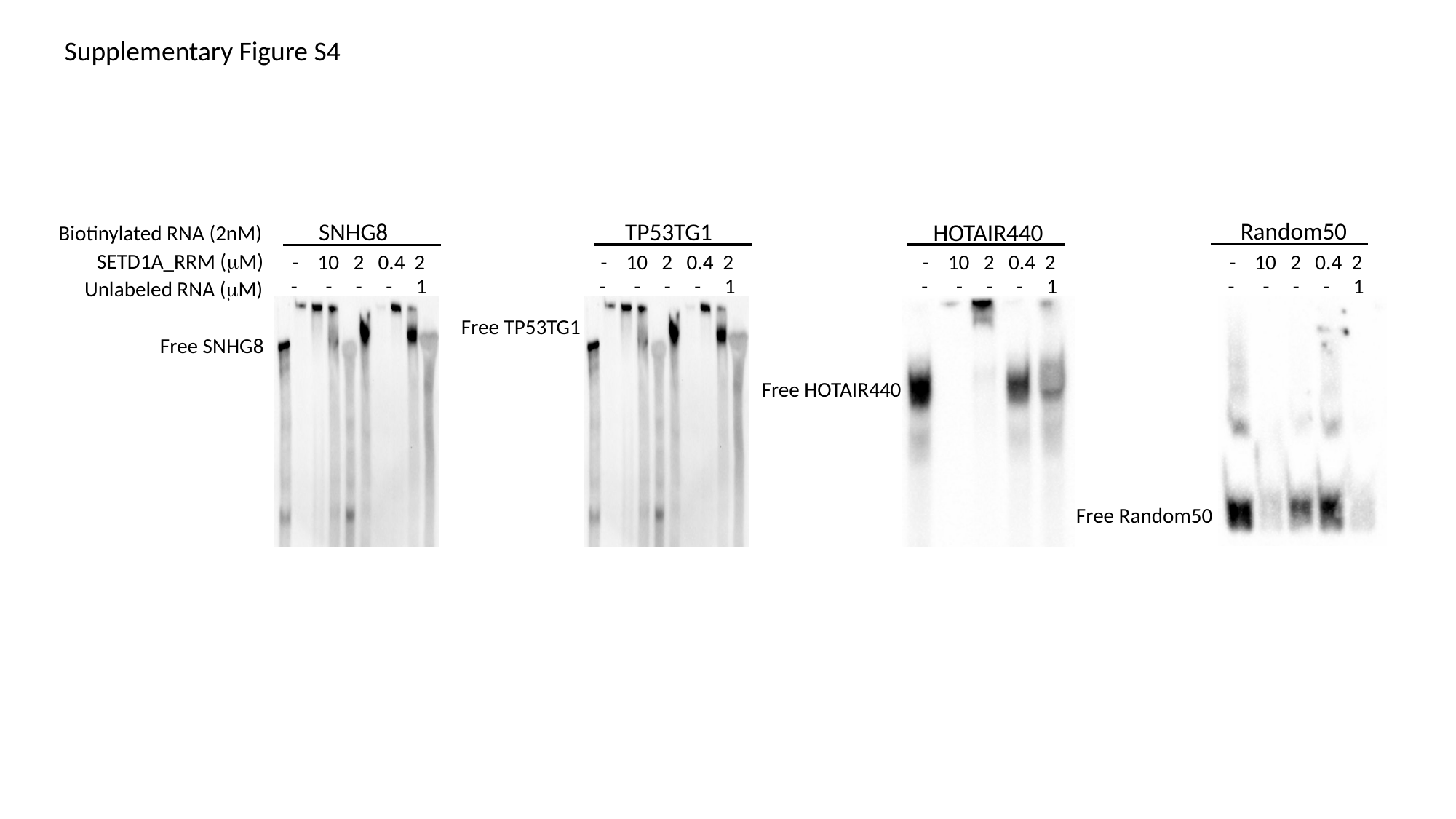

Supplementary Figure S4
Random50
SNHG8
TP53TG1
HOTAIR440
Biotinylated RNA (2nM)
SETD1A_RRM (mM)
- 10 2 0.4 2
- - - - 1
- 10 2 0.4 2
- - - - 1
- 10 2 0.4 2
- - - - 1
- 10 2 0.4 2
- - - - 1
Unlabeled RNA (mM)
Free TP53TG1
Free SNHG8
Free HOTAIR440
Free Random50

## Slide 5
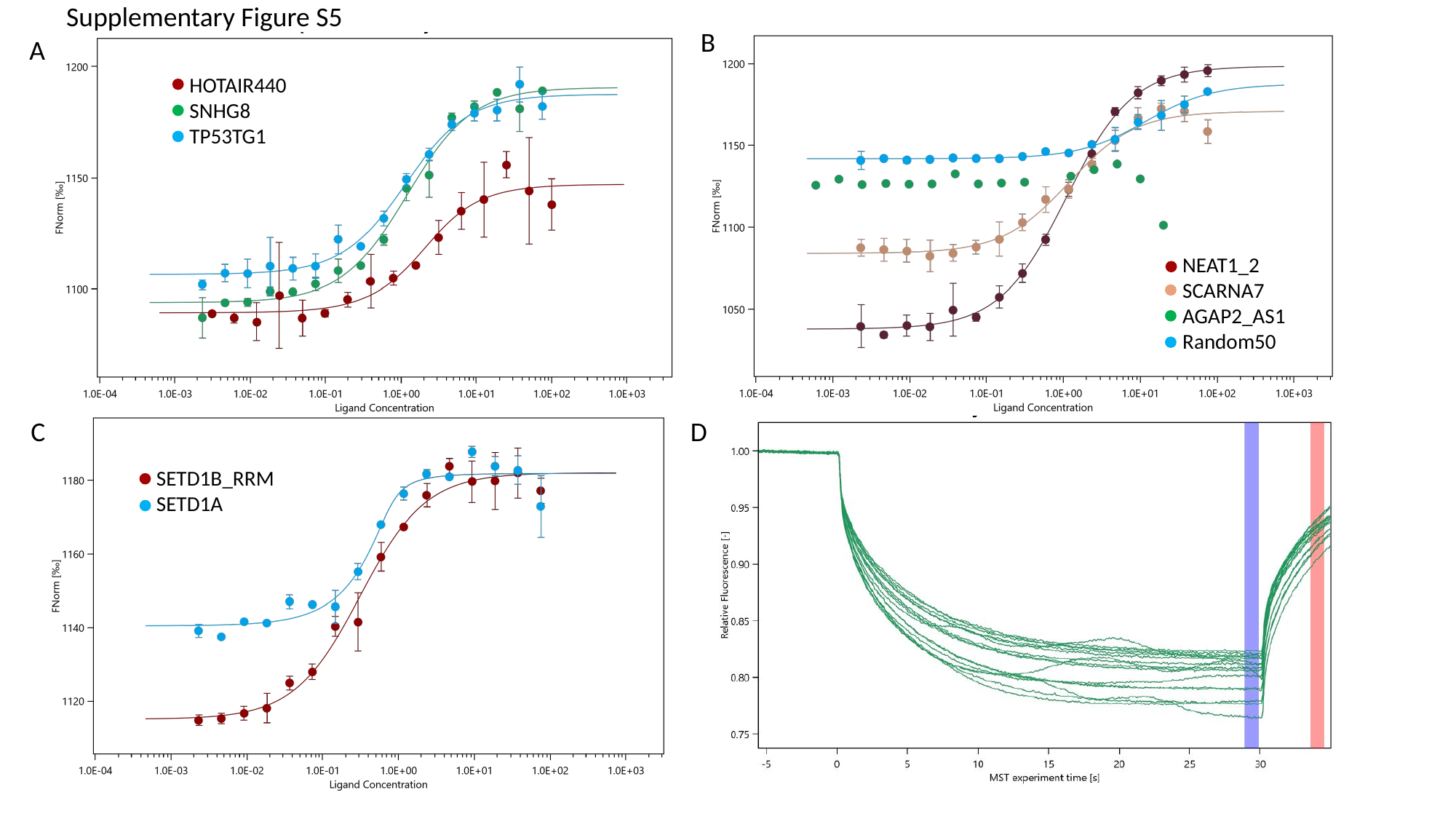

Supplementary Figure S5
B
A
HOTAIR440
SNHG8
TP53TG1
NEAT1_2
SCARNA7
AGAP2_AS1
Random50
C
D
SETD1B_RRM
SETD1A

## Slide 6
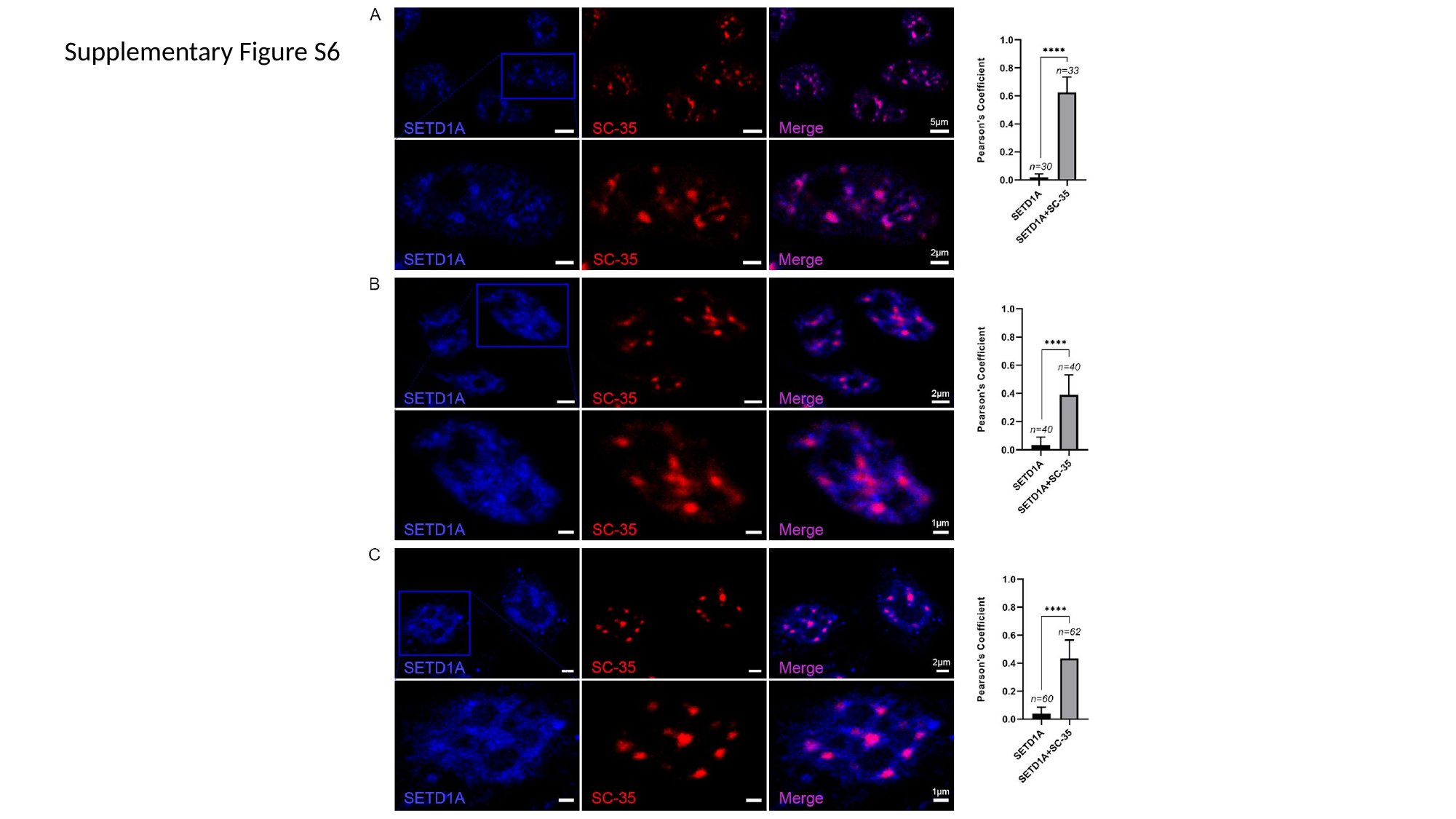

Supplementary Figure S6

## Slide 7
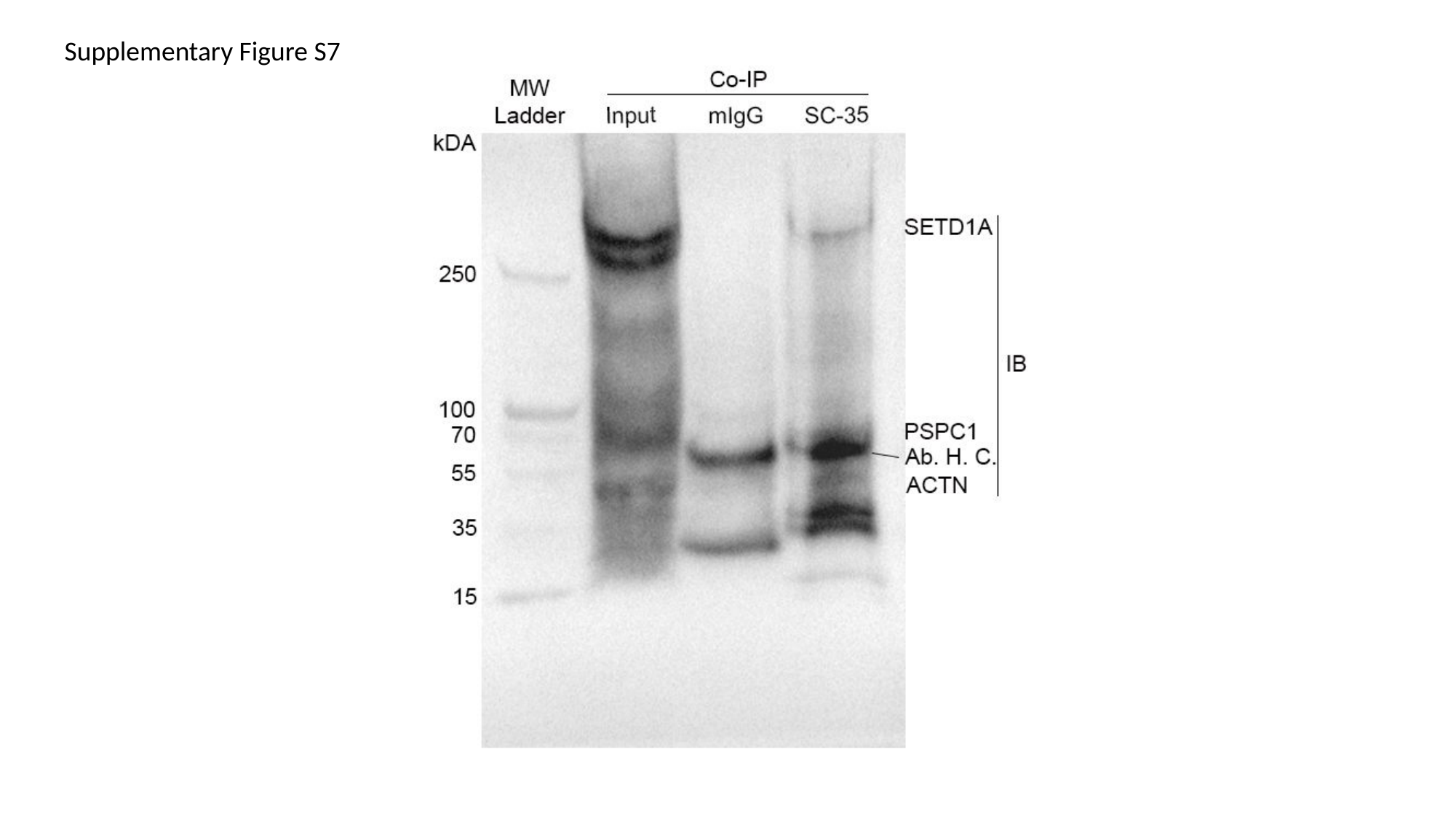

Supplementary Figure S7

## Slide 8
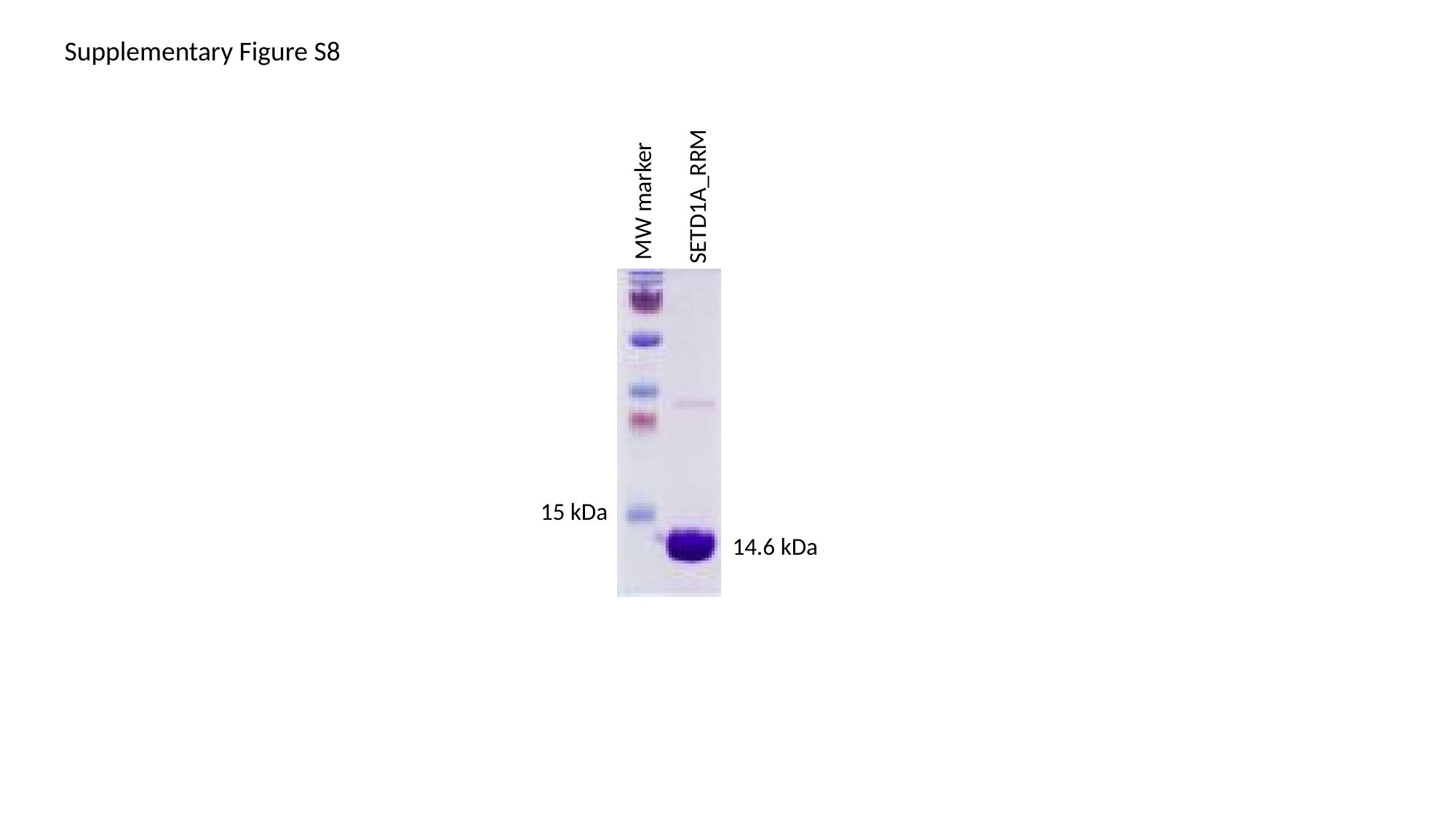

Supplementary Figure S8
SETD1A_RRM
MW marker
15 kDa
14.6 kDa

## Slide 9
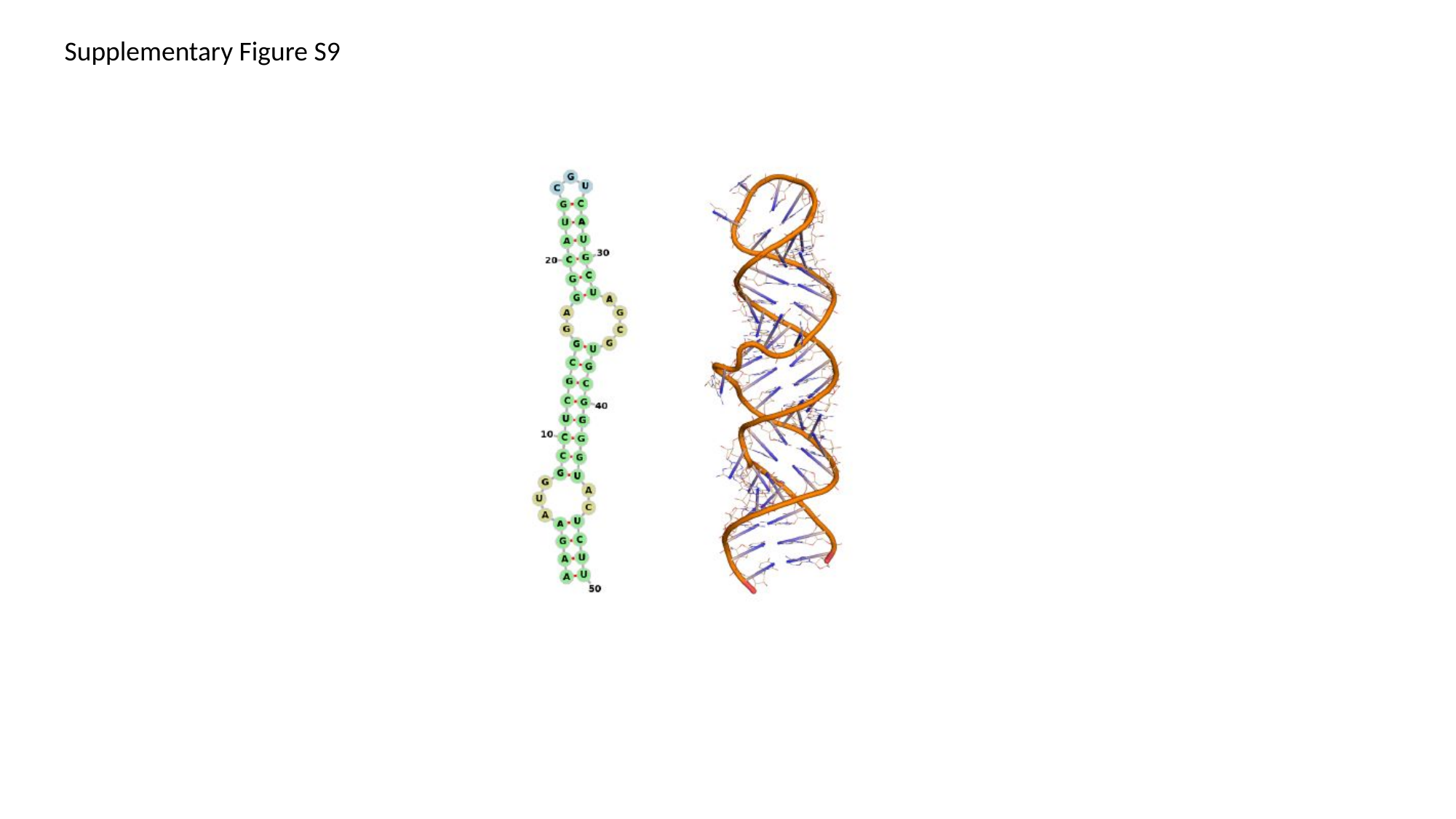

Supplementary Figure S9
